# Effects of classical PKC activation on hippocampal neurogenesis and cognitive performance: mechanism of action

**DOI:** 10.1038/s41386-020-00934-y

**Published:** 2020-12-17

**Authors:** Samuel Domínguez-García, Ricardo Gómez-Oliva, Noelia Geribaldi-Doldán, Carmen Hierro-Bujalance, Marta Sendra, Félix A. Ruiz, Livia Carrascal, Antonio J. Macías-Sánchez, Cristina Verástegui, Rosario Hernández-Galán, Mónica García-Alloza, Pedro Nunez-Abades, Carmen Castro

**Affiliations:** 1 Área de Fisiología, Facultad de Medicina, Universidad de Cádiz, Cádiz, Spain; 2 Instituto de Investigación e Innovación Biomédica de Cádiz (INiBICA), Cádiz, Spain; 3 Área de Nutrición, Facultad de Medicina Universidad de Cádiz, Cádiz, Spain; 4 Departamento de Fisiología, Facultad de Farmacia, Universidad de Sevilla, Sevilla, Spain; 5 Departamento de Química Orgánica, Facultad de Ciencias, Universidad de Cádiz, Puerto Real, Spain; 6 Departamento de Anatomía y Embriología Humanas, Facultad de Medicina, Universidad de Cádiz, Cádiz, Spain; 7 Present Address: Instituto de Investigaciones Marinas (IIM-CSIC), Vigo, Pontevedra Spain

**Keywords:** Cell signalling, Adult neurogenesis

## Abstract

Hippocampal neurogenesis has widely been linked to memory and learning performance. New neurons generated from neural stem cells (NSC) within the dentate gyrus of the hippocampus (DG) integrate in hippocampal circuitry participating in memory tasks. Several neurological and neuropsychiatric disorders show cognitive impairment together with a reduction in DG neurogenesis. Growth factors secreted within the DG promote neurogenesis. Protein kinases of the protein kinase C (PKC) family facilitate the release of several of these growth factors, highlighting the role of PKC isozymes as key target molecules for the development of drugs that induce hippocampal neurogenesis. PKC activating diterpenes have been shown to facilitate NSC proliferation in neurogenic niches when injected intracerebroventricularly. We show in here that long-term administration of diterpene ER272 promotes neurogenesis in the subventricular zone and in the DG of mice, affecting neuroblasts differentiation and neuronal maturation. A concomitant improvement in learning and spatial memory tasks performance can be observed. Insights into the mechanism of action reveal that this compound facilitates classical PKCα activation and promotes transforming growth factor alpha (TGFα) and, to a lesser extent, neuregulin release. Our results highlight the role of this molecule in the development of pharmacological drugs to treat neurological and neuropsychiatric disorders associated with memory loss and a deficient neurogenesis.

## Introduction

The generation of neurons from neural stem cells (NSC) is a complex process that occurs in mammals in brain regions referred to as neurogenic niches; mainly the dentate gyrus of the hippocampus (DG) and the subventricular zone (SVZ). NSC -or radial glial like cells in the DG- generate proliferative intermediate progenitors [1] in both niches, which have the capacity to generate slow proliferating neuronal progenitors (neuroblasts) and to a lesser extent glial progenitors, which differentiate into astrocytes or oligodendrocytes [2, 3]. Neuroblasts differentiate into mature neurons. Whereas granule cell neurons generated within the DG integrate into hippocampal circuits, SVZ neuroblasts migrate toward the olfactory bulb [4, 5] producing olfactory interneurons. DG neurogenesis has been linked to learning and memory [6, 7], and a number of different neurological and neuropsychiatric pathologies, such as depression, and response to stress [8, 9]. Alterations in the neurogenic rate and a reduction in the number of granule cells have been associated with depression and suicidal behavior [10, 11] whereas antidepressants promote hippocampal neurogenesis in rodents [12]. Neurogenesis is regulated by growth factors released within the niches such as neuregulin or transforming growth factor alpha (TGFα), which activate ErbB receptors within the DG and SVZ, promoting neurogenesis [13–15]. Interestingly, behavioral alterations have been found in individuals with disrupted neuregulin signaling [13] and cognitive improvement has been observed in mouse models of ischemia upon treatment with TGFα [15]. In this context, seeking for agents that modulate growth factor release would be a good strategy to facilitate the generation of new neurons and their contribution to circuit reformation and function recovery.

ErbB ligands such as amphiregulin or TGFα are released to the extracellular medium in a proteolytic reaction catalyzed by convertases of the A Disintegrin and Metalloproteinase (ADAM) family such as ADAM17 [16]. The selectivity of this enzyme for each ligand is determined by phosphorylation reactions catalyzed by kinases of the protein kinase C (PKC) family [17]. PKC consists on three subfamilies of kinases: the classical, the novel, and the atypical. Classical PKCα activated by phorbol-12-myristate-13-acetate (PMA) catalyzes the phosphorylation of TGFα, and amphiregulin precursors facilitating their shedding mediated by ADAM17 and releasing the soluble ligand outside the cell. Similarly, activation of novel PKCδ is required for ADAM17 mediated secretion of neuregulin. Overall, ADAM17 substrate specificity and selectivity is mediated by the activation of different PKC isozymes, thus playing a key role in the secretion of different types of growth factors [17–19].

Previous reports show that diterpene compounds with 12-deoxyphorbol structure isolated from plants of the Euphorbia genus, with a capacity to activate specific isozymes of PKC, promote proliferation of neural progenitors 3 days after direct intracerebroventricular administration [20]. The purpose of this work is to study the long-term effect of non-invasive administration of diterpenes on neurogenesis and cognitive performance, and understand their mechanism of action.

## Methods

### Reagents

Isolation and purification of the 12-deoxyphorbol ER272 (CAS:25090-74-8) was performed in our laboratory as described previously [20] (see supplementary methods). PKC inhibitors were purchased from Sigma-Aldrich (St. Louis, MO) and Calbiochem (Millipore, Billerica, MA). The general inhibitor of PKC bisindolylmaleimide I (Gö6850) and the classical PKC inhibitor (Gö6976) were added to cell cultures at final concentrations of 5 µM and 1 µM, respectively and were added 30 min before the addition of the PKC activators. Other products, unless otherwise indicated, were purchased from Sigma-Aldrich (St. Louis, MO, USA).

### Animals

SVZ derived neural progenitor cells (NPC) were isolated from 7-day postnatal CD1 mice and used for in vitro experiments. For in vivo experiments 2-month-old male CD1 mice were used. Animals were housed under controlled conditions of temperature (21–23 °C) and light (LD 12:12) with free access to food (AO4 standard maintenance diet, SAFE, Épinay-sur-Orge, France) and water. Care and handling of animals were performed according to the Guidelines of the European Union Council (2010/63/EU), and the Spanish regulations (65/2012 and RD53/2013) for the use of laboratory animals. All studies involving animals are reported in accordance with the ARRIVE guidelines for reporting experiments involving animals [21, 22]. The protocols used have been authorized by the Ethics Committee of the “Consejería de Agricultura, Pesca y Desarrollo sostenible” of the Junta de Andalucía”, Spain with the approval numbers 30/03/2016/038 and 04/03/2020/033.

### Intranasal administration of ER272

ER272, was delivered intranasally as previously described [23–25]. Treatments were administered manually while the animal was placed in a standing position with an extended neck as previously described [25]. 18 µL of each solution (5 µM ER272 in saline, or saline as vehicle) was delivered over both nasal cavities alternating 3 µL/each using a micropipette. Mouse was maintained in such position for 10 additional seconds to ensure all fluid was inhaled. In all experiments, mice were coded, treatment (vehicle or ER272) was assigned randomly to code numbers and applied. In addition, blind quantifications were performed to avoid subjective biases.

### ICV administration of ER272

Adult mice were anesthetized and placed on a stereotaxic frame to administrate ER272 (1 µM), the general PKC inhibitor Gö5068 (1 μM), a combination of ER272 (1 μM) and Gö5068 (1 μM) or vehicle as previously described [20, 26] (see supplementary methods).

### Brain processing and immunohistochemistry

At the end of the treatment brains were perfused with paraformaldehyde (PFA) and sliced using a cryotome into 30 µm sections. Immunohistochemistry was performed as previously described [20, 26, 27]. See antibodies in Supplementary Tables 1 and 2.

### Quantification of neurogenesis in brain sections

Cells positive for BrdU, DCX, GFAP, EGFR, nestin, or Ascl1 in the SVZ and DG were estimated as described [28, 29]. Positive cells were counted throughout the entire DG area or lateral and laterodorsal walls of the lateral ventricles in every fifth section; 14–16 sections per brain where analyzed under fluorescence microscopy at ×20 magnification. Mice were coded depending on the treatment and quantification of cells in brain slices was done in blinded analysis. Cells in the SVZ and DG of both brain hemispheres have been quantified unless otherwise indicated.

### Quantitative analysis of neuronal morphology

Manual quantitative analysis of BrdU^+^/ß-III-tubulin^+^ neurons within the DG of the mouse hippocampus was performed as described in Henley et al., 2019 [30]. Fluorescence microscopy images of ß-III-tubulin^+^/BrdU^+^ cells were imported into ImageJ software to quantify cell area and length. Cell and dendritic area were manually measured using “polygon selections” tool on ImageJ software, tracing the cell soma and each neurites. Only projected ß-III-tubulin^+^ neurites from BrdU^+^ somas were taken into account. Length of the dendritic arbors of neurons was manually measured using the “freehand line” tool on ImageJ software, indicating the start and end of each dendrite.

### SVZ-derived NPC isolation and culture

SVZ- derived cells were isolate following the same procedure described in Rabaneda et al., 2008 [28]. For in vitro experiments in proliferation conditions, growth factors EGF and bFGF were used as indicated in the figure legends (see supplementary methods).

### Neurosphere assay

The effect of ER272 was tested by neurosphere assays in order to evaluate NPC proliferation. ER272 was added at the time of seeding. All experiments were run by triplicates a minimum of five independent times. Number and size of neurospheres were measured as previously described [28, 29] using the software ImageJ (see supplementary methods).

### Neurosphere treatment and transfection

Neurosphere cells were transfected with specific siRNA SmartPool One target siRNA from Horizon (Cambridge, UK) against each specific PKC isozyme using Lipofectamine 2000, following the manufacturer’s instructions as previously described [26]. ER272 was added and cells were maintained for 48 additional hours before being used for flow cytometry.

### Flow cytometry

For proliferation studies, cells were disaggregated from the neurospheres and fixed in 4% PFA rinsed with PBS and centrifuged (300 × *g*; 5 min). Cells were incubated in a blocking solution (1 mg mL^−1^ bovine serum albumin and 0.3% Triton X100) followed by an incubation with fluorescent antibody (Supplementary Table S1). Cells were then rinsed in PBS, resuspended in FACS buffer and analyzed in a Attune NxT flow cytometer (Invitrogen).

### HEK293T culture, cloning and transfection

HEK293T obtained from ATCC (Manassas, VA, USA) were cultured and transfected as previously described [31]. After an overnight incubation, cells were left for 30 min in serum-free Fluorobrite DMEM (Thermo Fisher Scientific) and used either in time-lapse or fluorescence experiments (see supplementary methods).

### Cloning of human neuregulin and TGFα cDNA fused to GFP and Cherry

Full-length cDNA encoding the membrane-bound isoform of human pro-neuregulin-1 β1-type (NRG1, NCBI reference sequence: NP_039250.2) with mCherry cDNA inserted between nucleotides 93 and 94 of NRG1 open reading frame was cloned into pEGFP-N1 to add EGFP cDNA to the 3′ end. Construct was synthesized by GeneCust (Boynes, France) to generate the mCherry-NRG1-GFP construct. The mCherry-TGFα-GFP construct containing the human transforming growth factor alpha (TGFA, NCBI reference sequence: NM_003236.4), containing mCherry cDNA between nucleotides 126 and 127 of TGFA was built using the same strategy and synthesized by GeneCust (Boynes, France).

### Time lapse experiments and fluorescence analysis of mCherry-fused TGFα or neuregulin in the culture medium of HEK293T

HEK293T were plated in µ–dishes (35 mm high, Ibidi, Munich, Germany). Cells were treated with ER272 or inhibitors, and images were taken as described in figure legends. For fluorescence measurements, 200000 HEK293T cells were plated in 1 mL of medium Costar^®^ 12-well cell culture microplate and fluorescence in the medium was measured as described in figure legend (additional information in supplementary methods).

### Morris water maze (MWM)

Spatial memory and learning tasks were analyze starting 10 days before sacrifice using the MWM test in control and treated mice the 1 day after the NOD test was concluded as previously described [32] and shown in figure legend (additional information in supplementary methods).

### Motor activity and new object discrimination (NOD) task

Motor activity was analyzed measuring the distance travelled by each mouse during a 30 s period before initiating the NOD test. Then integrated episodic memory for the paradigms “what”, “when” and “where” was analyzed as described in previous reports [32] (additional information in supplementary methods). In brief, on the first sample trial, mice were placed into the center of the box containing three copies of a novel object (yellow balls) arranged in a triangle-shaped spatial configuration and allowed to explore them for 5 min. After a delay of 30 min, the mice received a second sample trial with four novel objects (navy cubes), arranged in a quadratic-shaped spatial configuration, for 5 min. After a delay of 30 min, the mice received a test trial with two copies of the object from sample trial 2 (“recent” objects) placed at the same position, and two copies of the object from sample trial 1 (“familiar” objects) placed one of them at the same position (“old non-displaced” object) and the another in a new position (“familiar displaced” object). Integrated episodic memory for “what”, “where” and “when” was analyzed as previously described [33–35]: “What” was defined as the difference in time exploring familiar and recent objects, “where” was defined as the difference in time exploring displaced and non-displaced objects and “when” was defined as the difference between time exploring familiar non displaced and recent non displaced objects.

### Statistical analysis

The data and statistical analysis comply with the recommendations on experimental design and analysis in pharmacology [36]. Statistical analysis was performed using the computer program IBM SPSS Statistics 22. Unless otherwise indicated, normal distribution of the data was first analyzed using a Shapiro–Wilks test. Then, a Brown Forsythe test was performed to test the equality of variances. Afterwards, when more than one treatment group were compared, statistical analyses were performed using one-way ANOVA followed by a post-hoc Bonferroni’s test unless otherwise indicated. Two way-ANOVA (group × day) was used in the acquisition phase of the MWM. A Student’s *t* test was used when only one treatment group was compared with the control. Differences were considered significant at values of *p* < 0.05. In general, sample size used in statistical analysis were *n* = 6–10 for in vivo experiments and *n* = 5–9 for in vitro experiments. Sample sizes were chosen based on previous works related to this one [26, 29, 31, 37].

## Results

### Short term intranasal administration of ER272 promotes proliferation of in the DG and SVZ

**Fig. 1:**
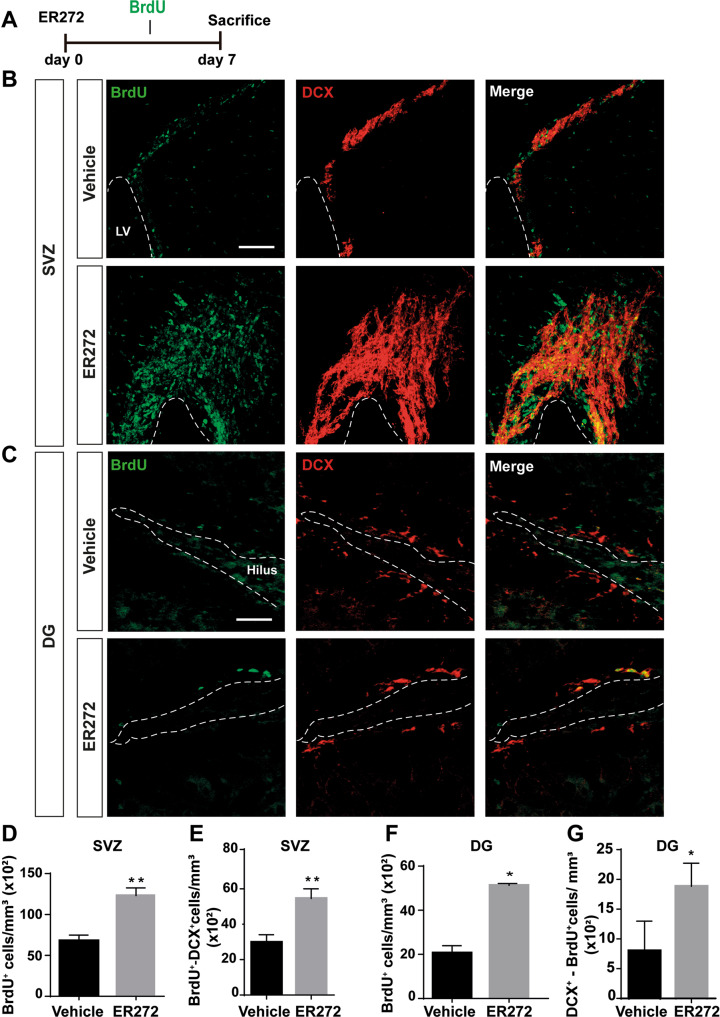
Intranasal administration of ER272 promotes neurogenesis in the SVZ and the DG. Intranasal ER272 (1 µM) or only vehicle was administered during 7 days to healthy 2-month-old adult mice. **A** Scheme of BrdU administration. All mice were intraperitoneally-injected with BrdU (100 mg/kg) during 7 days every 2 days as described in methods. **B** Representative confocal microcopy images of the SVZ of adult mice treated with ER272 or only vehicle. Scale bar = 50 µm. The dotted lines indicate lateral ventricle limits (LV). Slices were processed for the immunohistochemical detection the proliferation marker BrdU and the neuroblast marker doublecortin (DCX). **C** Representative confocal microcopy images of the DG of the hippocampus of adult mice treated with ER272 or only vehicle. Scale bar = 50 µm. The dotted lines indicate the DG limits. Slides were processed for the immunohistochemical detection the proliferation marker BrdU and the neuroblast marker doublecortin (DCX). **D** Graph shows the number of proliferating cells marked with BrdU per mm^3^ in the SVZ of the indicated animal groups. Data are the means ± S.E.M of six animals *n* = 6. Statistical analysis: **p = 0.0033 in two tailed unpaired Student’s *t* test comparing ER272 treatment with the control group (treated only with vehicle). **E** Graph shows the number of BrdU^+^ cells that co-express the neuroblast marker DCX (doublecortin) in the SVZ per mm^3^. Data are the means ± S.E.M of six animals *n* = 6. Statistical analysis: *p = 0.0084 in two tailed unpaired Student’s *t* test comparing ER272 treatment with the control group (treated only with vehicle). **F** Graph shows the number of proliferating cells marked with BrdU per mm^3^ in the DG of the hippocampus of the indicated animal groups. Data are the means ± S.E.M of six animals *n* = 6. Statistical analysis: ***p = 0.0006 in two tailed unpaired Student’s *t* test comparing ER272 treatment with the control group (treated only with vehicle). **G** Graph shows the number of BrdU^+^ cells that co-express the neuroblast marker DCX (doublecortin) in the DG of the hippocampus per mm^3^. Data are the means ± S.E.M of six animals *n* = 6. Statistical analysis: *p = 0.0139 in two tailed unpaired Student’s *t* test comparing ER272 treatment with the control group (treated only with vehicle).

Mice given ER272 for 7 days by intranasal administration and receiving BrdU every 2 days since the first day of treatment (Fig. 1A), showed a higher number of cells that incorporated BrdU over the course of the treatment compared to controls, in the SVZ (Fig. 1B, D) and DG (Fig. 1C, F). In addition, ER272 treatment increased the number of DCX^+^ neuroblasts that had incorporated BrdU in both niches (Fig. 1B, C, E, G). No effect was observed of ER272 on the number of BrdU^+^/GFAP^+^ cells within the SVZ. In the DG, we observed a reduction in the number of GFAP^+^ cells that incorporated BrdU (Supplementary Fig. S1). However, no effect was observed in the DG regarding the number of mature glial cells (Supplementary Fig. S2).

The number of Ascl1^+^ transit amplifying cells in the SVZ increased in treated mice, whereas no statistically detectable alterations in the number of Ascl1^+^ cells or in the percentage of Ascl1^+^/BrdU^+^ of the total BrdU^+^ cells were observed in the DG (Supplementary Fig. S3).

### Long-term intranasal administration of ER272 promotes neurogenesis in the DG

**Fig. 2:**
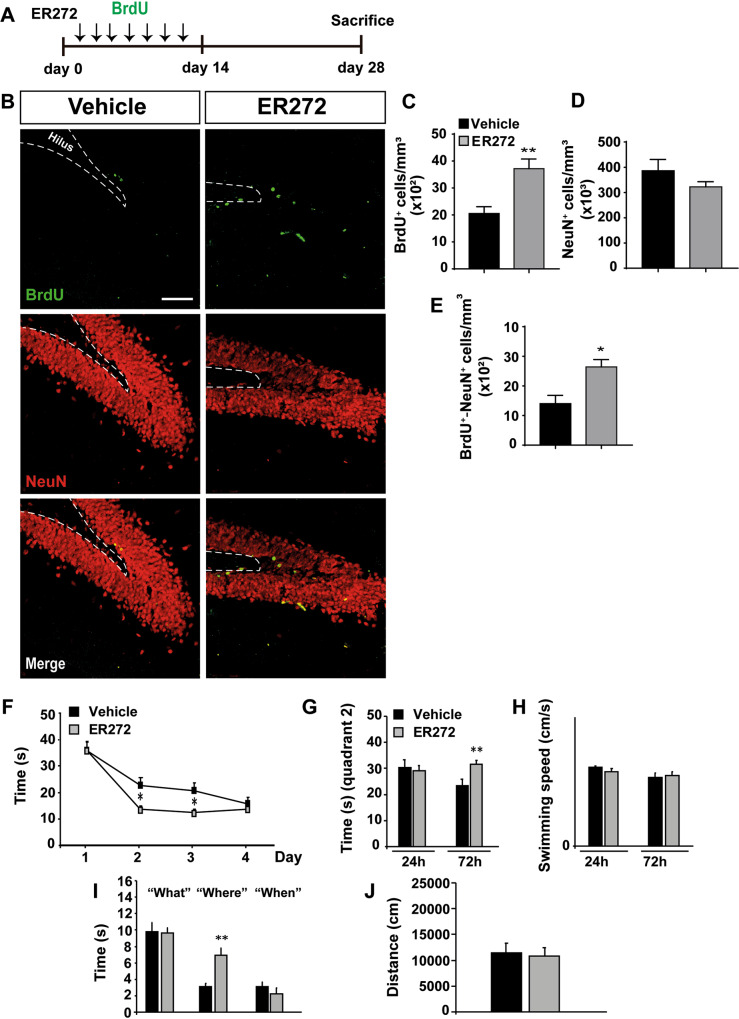
Long term intranasal administration of ER272 promotes neurogenesis in the DG and enhances learning and memory. Intranasal ER272 (1 µM) or only vehicle was administered during 28 days to healthy 2-month-old adult mice. **A** Scheme of BrdU administration. All mice were intraperitoneally-injected with BrdU (100 mg/kg) during the first 14 days every 2 days as described in methods. **B** Representative confocal microcopy images of the DG of adult mice hippocampus treated with ER272 or only vehicle. Scale bar = 50 µm. The dotted lines indicate DG limits. Slices were processed for the immunohistochemical detection the proliferation marker BrdU and the neuronal marker NeuN. **C** Graph shows the number of proliferating cells marked with BrdU per mm^3^ in the DG of the indicated animal groups. Data are the means ± S.E.M of ten animals *n* = 10. Statistical analysis: *p = 0.0270 in two tailed unpaired Student’s *t* test comparing ER272 treatment with the control group (treated only with vehicle). **D** Graph shows the percentage of NeuN^+^ cells per mm^3^ in the DG of the indicated treated groups. Data are the means ± S.E.M of six animals *n* = 6. Statistical analysis: *p = 0.034 in two tailed unpaired Student’s *t* test comparing ER272 treatment with the control group (treated only with vehicle). **E** Graph shows cells that co-express BrdU and the neuronal marker NeuN per mm^3^ in the DG of the hippocampus. Data are the means ± S.E.M of ten animals *n* = 10. Statistical analysis: *p = 0.040 in two tailed unpaired Student’s *t* test comparing ER272 treatment with the control group (treated only with vehicle). **F** Scape latency in the MWM test during the 4 day acquisition phase. We did not detect a significant treatment × day effect, when we compared the groups under study [*F*_(3, 290)_ = 1.61 *p* = 0.186]. Further assessment on individual days revealed that ER272 can significantly reduce the time required to find the platform: day 1 (*p* = 0.972); day 2 (*p = 0.011 vs. control; statistical power 0.728), day 3 (*p = 0.016 vs. control; statistical power 0.683), day 4 (*p* = 0.460). Differences were detected by Student’s *t* test for independent samples. **G** Time spent in quadrant 2 during the retention phase 24 and 72 h after the acquisition test. ER272 treatment improved performance in the retention phase of the MWM. While no differences were detected among groups in the retention phase, 24 h after acquisition (*p* = 0.731) in the time spent where the platform used to be located (quadrant 2), ER272 increased the time in quadrant 2, 72 h after completing the acquisition phase (**p = 0.009 vs. Control). Differences detected by Student’s *t* test for independent samples. **H** Swimming speed was not affected by the treatments in the 24 h (*p* = 0.539) or 72 h (*p* = 0.716) retention phase. Differences detected by Student’s *t* test for independent samples. **I** Episodic memory was improved in the new object discrimination test after ER272 administration. Whereas no differences were detected for parading “what” (*p* = 0.9333) or “when” (*p* = 0.319), performance for paradigm “where” was significantly improved after ER272 treatment (**p < 0.01 vs. Control). Differences were detected by Student *t* test for independent samples. **J** Graph representing motor activity. No differences were detected (*p* = 0.820) using Student’s *t* test for independent samples.

The effect of 4 weeks treatment with ER272 on DG neurogenesis was tested in mice that received BrdU during the first 2 weeks of treatment (Fig. 2A). As shown in Fig. 2, ER272 increased the number of BrdU^+^ cells within the DG (Fig. 2B, C) as well as the number of mature NeuN^+^ neurons that had incorporated BrdU (Fig. 2B, E) (Fig. 2B, D). An elevated number of BrdU/DCX^+^ cells in the DG after 28 days of treatment was detected, however the total number of DCX^+^ cells remained unaltered (Supplementary Fig. S4, A-C). Also, we performed a pilot study to analyze whether ER272 affected the morphology of newly generated neurons. The morphological analysis of BrdU^+^/β-III-tubulin^+^ neurons, reconstructed as previously described [30], was performed quantifying the soma area, cell area, maximum neurite length (maximum distance from soma to a terminal ending/neuron), and the total neurite length calculated as the summed length of all branches of a neuron. Quantification was performed according to previous works [38, 39] in BrdU^+^ neurons detected as BrdU^+^/β-III-tubulin^+^. Results show no differences in any of these parameters in mice treated with ER272 compared to control. Thus, no alterations on the morphology of newly generated neurons detected as BrdU^+^/β-III-tubulin^+^ were induced by ER272 treatment (Supplementary Fig. S5).

### Long-term intranasal administration of ER272 improves cognitive performance

We next analyzed spatial and episodic memory using the MWM and NOD tasks. In the MWM control and treated mice progressively reduced the time used to find the platform from day 1 to day 4, along the acquisition phase. Although differences between both groups were found during the training phase on days 2 and 3, no treatment × day differences were observed (Fig. 2F) indicating no effect of the treatment on the training phase. In the retention phase of the MWM, mice treated with ER272 improved performance. No differences in the time spent in quadrant 2, where the platform used to be located (quadrant 2) were detected among groups in the retention phase 24 h after acquisition, however, ER272 increased the time in quadrant 2, 72 h after completing the acquisition phase (Fig. 2G). Swimming speed was not affected by the treatments neither in the 24 h or 72 h retention phases (Fig. 2H).

Episodic memory was analyzed by the NOD test, finding an improvement in mice treated with ER272. No differences were detected for paradigm “what” (*p* = 0.9333) or “when” (*p* = 0.319), however performance for paradigm “where” was significantly improved after ER272 treatment (*p* < 0.01 vs. control) (Fig. 2I). No differences were found in motor activity measured as the distance moved by these mice (Fig. 2J). These results indicated an improvement in cognitive performance, particularly in spatial memory.

### ER272 mimics the effect of EGF

**Fig. 3:**
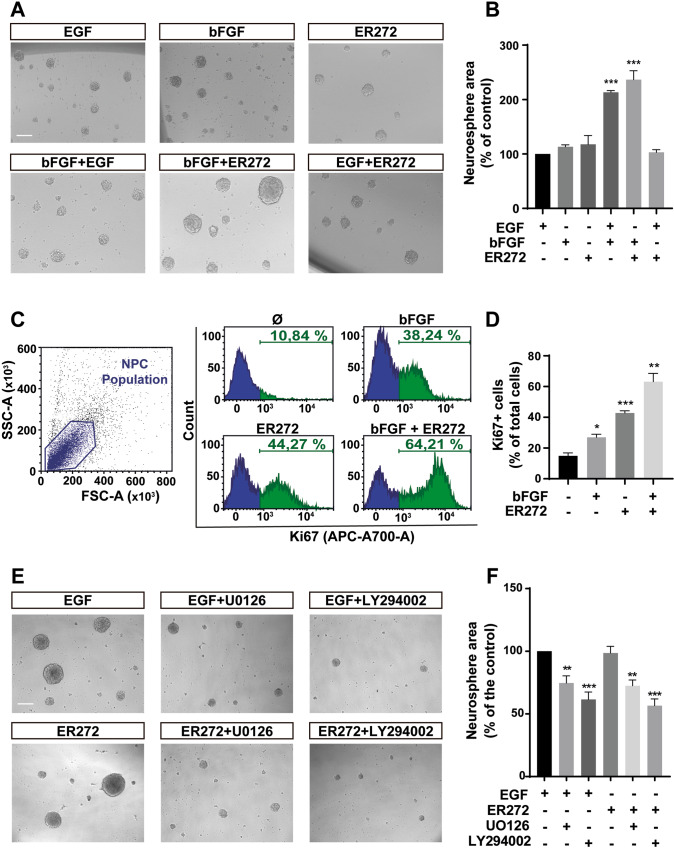
ER272 mimics the proliferative effect of the epidermal growth factor in vitro. **A** Representative phase-contrast microscopy images of neurospheres cultured for 72 h in presence or absence of the indicated growth factors and ER272. Scale bar indicates 100 µm. **B** Graph shows the effect of the different treatments on neurospheres area expressed as the percentage of control (only EGF treatment). Data shown are the mean ± S.E.M. of nine independent measurements. Statistical analysis: ***p < 0.0001 in two tailed unpaired Student’s *t* test comparing EGF with EGF + bFGF and with bFGF+ER272. **C** Flow cytometry analysis of cells isolated from neurospheres treated under the different conditions indicated and labelled with anti-Ki67 antibody; left panel: forward/side scatter; central panel: cell count vs. fluorescence intensity in the four different condition; right panel graph shows the percentage of cells labelled with Ki67 in the four different conditions. **D** Quantification of proliferating cells (Ki67^+^) in flow cytometry plots as those shown in (**C**). Statistical analysis: *p = 0.0108 in two tailed unpaired Student’s *t* test comparing control with bFGF; ***p = 0.0003 comparing control with ER272; **p = 0.0011 comparing control with bFGF+ER272. **E** Representative phase-contrast microscopy images of neurospheres treated for 72 h with EGF or ER272 and the MAPK inhibitor U0126 (10 μM) or the PI3-K inhibitor LY294002 (50 μM). Inhibitors were added 30 min before EGF or ER272. Scale bar = 100 μm. **F** Graph shows the effect of the different treatments on neurospheres area expressed as the percentage of control (only EGF treatment). Data shown are the mean ± S.E.M. of nine independent measurements. Statistical analysis: **p = 0.0128 in two tailed unpaired Student’s *t* test comparing EGF with EGF + U0126. ***p < 0*.*0001 in two tailed unpaired Student’s *t* test comparing EGF with EGF and with EGF + LY294002. **p = 0.0002 in two tailed unpaired Student’s *t* test comparing ER272 with ER272 + U0126. ***p < 0.0001 in two tailed unpaired Student’s *t* test comparing EGF with ER272 and with ER272 + LY294002.

To study ER272 mechanism of action, we examined the capacity of ER272 to mimic the effect of EGFR ligands such as EGF in cultures using the neurosphere assay. As shown in Fig. 3A, B, the size of the neurospheres in cultures treated with either EGF or bFGF was similar, whereas in the presence of both growth factors (EGF + bFGF), the effect was additive. However, the neurosphere size in cultures stimulated with ER272 was larger than that of cultures stimulated with only one of the growth factors, and a combination of bFGF + ER272 increased the size of the neurosphere more than the combination of EGF + bFGF. On the contrary, the addition of ER272 to EGF stimulated cultures did not induce an increase in neurosphere size. These results suggested that ER272 mimicked the effect of EGF in cultures by either directly activating the receptor or by inducing the release of EGFR ligands. Similarly, detection of Ki67 using flow cytometry in cells from neurosphere cultures revealed that ER272 increased the number of Ki67^+^ cells, being more potent than bFGF. The effects of bFGF and ER272 were additive, supporting the hypothesis that ER272 facilitates EGFR stimulation (Fig. 3C, D). The additive effect of ER272 was not specific of bGFG. Like EGF, ER272-induced a proliferative effect even in the presence of another different neurotrophic factor like BDNF (20 ng/mL) (Supplementary Fig. S6). Neurospheres cultured only in the presence of BDNF show a smaller size compared to those cultured in the presence of EGF or ER272. The addition of both EGF or ER272 to cultures grown with BDNF increased the size of the neurospheres in a similar manner.

In order to further study the similarities between the effect of EGFR ligands and ER272 we ruled out the effect of ER272 on activating two signaling pathways initiated by EGF: the MAPK and PI_3_K–AKT pathways. Thus, using the neurosphere assay we analyzed the effect of ER272 on neurosphere cultures in the presence of inhibitors of both pathways (10 μM of U0126 and 50 μM of LY94002 respectively). As shown in Fig. 3E, F, the effect of both EGF and ER272 on neurosphere size was abolished by the addition of both inhibitors, indicating the role of these two pathways in the ER272-mediated proliferative effect.

We then investigated whether the administration of ER272 in vivo induced proliferation of EGFR^+^ cells. Our results show that a single ICV injection of ER272 in mice that received BrdU during 3 days after the injections, increased the number of BrdU^+^/EGFR^+^/nestin^+^ undifferentiated progenitors in the SVZ 3 days post injection (Supplementary Fig. S7), thus supporting the hypothesis that ER272 facilitated the release of EGFR ligands.

### The proliferative effect of ER272 is mediated by classical PKCα activation

In order to demonstrate that the effect of ER272 on NPC proliferation in vivo was dependent on PKC activation, we tested the effect of a single ipsilateral ICV injections of ER272 (1 μM) in mice in combination with the general PKC Gö6850 (1 μM). As expected, 3 days after ICV injections of ER272 the total number of BrdU^+^ and BrdU^+^/DCX^+^ cells in the ipsilateral and contralateral SVZ and DG increased compared to that of untreated mice, whereas the PKC inhibitor significantly reduced this number. The addition of ER272 in combination with the general PKC inhibitor abolished the proliferative effect in both the SVZ and DG (Supplementary Fig. S8).

**Fig. 4:**
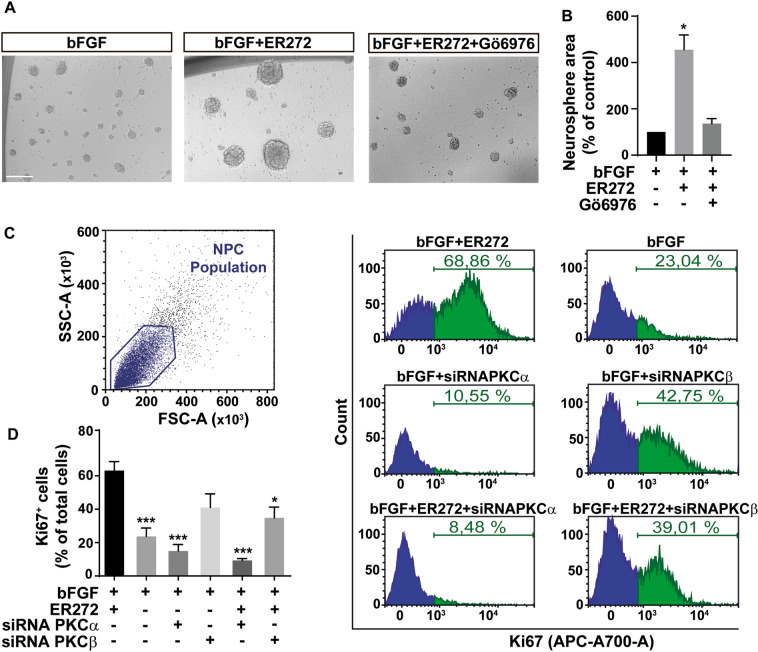
ER272 induces proliferation via classical PKC activation in NPC cultures. **A** Representative phase-contrast microscopy images of neurospheres cultured for 72 h in presence or absence of the basic fibroblastic growth factor (bFGF), ER272 or the classical PKC inhibitor (Gö6976). Scale bar indicates 100 µm. **B** Graph shows the effect of the different treatments on neurospheres area expressed as the percentage of control (bFGF treatment). Data shown are the mean ± S.E.M. of nine independent measurements. Statistical analysis: *p = 0.037 in two tailed unpaired Student’s *t* test comparing bFGF with bFGF+ER272. **C** Flow cytometry analysis of cells isolated from neurospheres treated under the different conditions indicated and labelled with anti- Ki67 antibody; left panel: forward/side scatter; right panel: cell count vs. fluorescence intensity in the six different conditions indicated. **D** Quantification of proliferating cells (Ki67^+^) in flow cytometry plots as those shown in (**C**). Statistical analysis: one-way ANOVA test: ***p = 0.0005, bFGF + ER272 vs. bFGF; ***p = 0.0004 bFGF + ER272 vs. bFGF + siRNA α; ***p < 0.0001 bFGF + ER272 vs. bFGF + ER272 + siRNA α; *p = 0.0165, bFGF + ER272 vs. bFGF + ER272 + siRNA β.

We next examined the PKC subfamily responsible for the proliferative effect of ER272 using the neurosphere assay. The size of the neurospheres in cultures treated with ER272 was larger than that of control cultures (Fig. 4A, B). This effect on neurosphere size was reverted by a classical PKC inhibitor (Fig. 4A, B).

It was next analyzed the effect of ER272 on NPC proliferation after cells were transfected with siRNAs to block the two classical PKC isozymes expressed in neurosphere cultures: PKCα PKCβ [26]. Proliferation was tested by analyzing the expression of Ki67 using flow cytometry (Fig. 4C, D). We observed an increase in the percentage of Ki67^+^ cells in cultures treated with ER272 (Fig. 4C, D). The effect was not observed in cultures of cells treated with ER272 and transfected with PKCα siRNA. Also, a smaller effect was observed in cultures of ER272 treated cells transfected with PKCβ siRNA.

### ER272 facilitates TGFα and neuregulin release

**Fig. 5:**
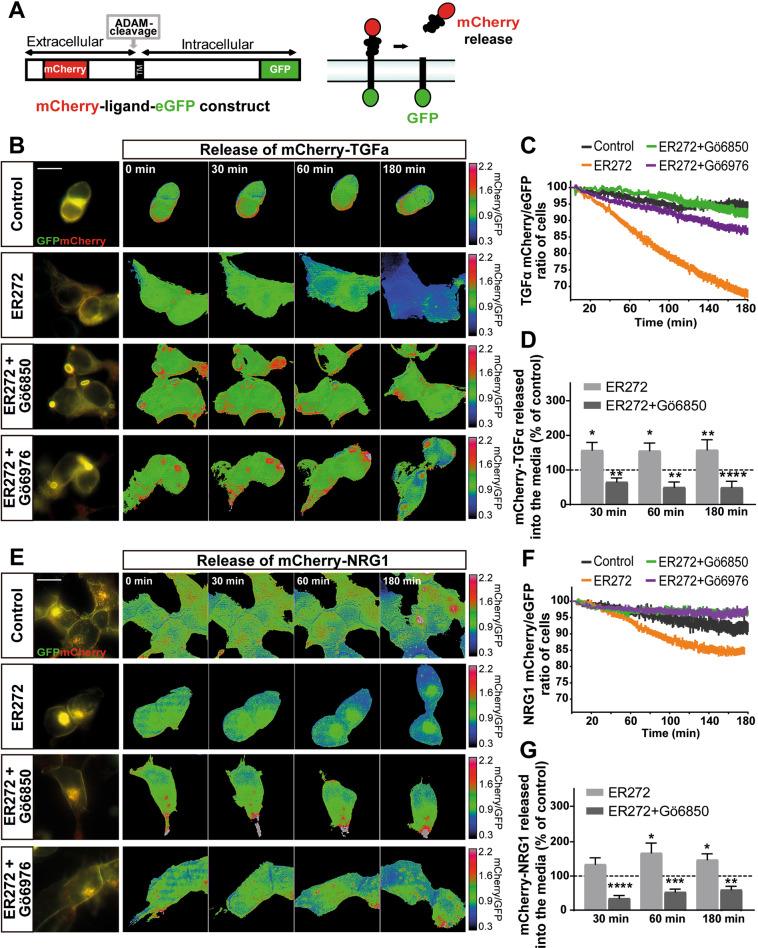
ER272 induces of mCherry-TGFα-GFP ectodomain shedding. **A** Scheme of mCherry-TGFα-GFP construct and action mechanism. **B** Images of mCherry-TGFα-GFP expressing, serum-starved HEK293T cells stimulated with diluent, ER272 (5 µM), ER272 plus the general PKC inhibitor Gö6850 (5 µM) and ER272 (5 µM) plus the classical inhibitor of PKC Gö6976 (1 µM) during 180 min. PKC inhibitors were added 30 min before ER272. mCherry/GFP ratio images at the indicated time points are shown in the intensity modulated display-mode. The color ranging goes from red to blue to represent mCherry/GFP ratios. The upper and lower limits of the ratio range are shown on the right. Scale bar represent 20 µm. **C** Quantitative analysis of the microscopic images obtained from the time-lapse assays of HEK293T cells expressing mCherry-TGFα-GFP. mCherry/GFP ratios were normalized to the average mCherry/GFP ratio measured before stimulation. The mean normalized mCherry/GFP ratios and SEM are shown, *n* = 40. See also supplementary movie 1 and 2. **D** Fluorescence analysis of m-Cherry signal in the culture medium of HEK293T cells transfected the mCherry-TGFα-eGFP construct. mCherry signal released to the culture media at different time points was measured in a fluorimeter using non-transfected cells as blank. 50 µL of medium (5% of the total volume) of control (transfected non-treated cells) and cells treated with ER272 and ER272 + Gö6850 were measured at different time points after the treatment. mCherry fluorescence was plotted as a ratio of the eGFP fluorescence measured in the cell fraction of control (dashed line) and treated cells (bars). Data are the mean values ± S.E.M of nine independent measurements (*n* = 9). Statistical analysis: two tailed unpaired Student’s *t* test (*ER272 vs. control at 30 min, *p* = 0.0191; **ER272 + Gö6850 vs. control at 30 min, *p* = 0.0049; *ER272 vs. control at 60 min, *p* = 0.0135; **ER272 + Gö6850 vs. control at 60 min, *p* = 0.0045; **ER272 vs. control at 180 min, *p* = 0.0024; ****ER272 + Gö6850 vs. control at 180 min, *p* < 0.0001). **E** Images of mCherry-NRG-GFP expressing, serum-starved HEK293T cells stimulated with diluent, ER272 (5 µM), ER272 plus the general PKC inhibitor Gö6850 (5 µM) and ER272 (5 µM) plus the classical inhibitor of PKC Gö6976 (1 µM) during 180 min. PKC inhibitors were added 30 min before ER272. mCherry/GFP ratio images at the indicated time points are shown in the intensity modulated display-mode. The color ranging goes from red to blue to represent mCherry/GFP ratios. The upper and lower limits of the ratio range are shown on the right. Scale bar represent 20 µm. **F** Quantitative analysis of the microscopic images obtained from the time-lapse assays of HEK293T cells expressing Cherry-NRG-GFP. mCherry/GFP ratios were normalized to the average mCherry/GFP ratio measured before stimulation. The mean normalized mCherry/GFP ratios and SEM are shown, *n* = 40. See also supplementary movie 3 and 4. **G** Fluorescence analysis of shedded mCherry-fused NRG. mCherry released to the culture media of transfected HEK293T cells was measured in a fluorimeter as described in the Material and Methods section. Cells were treated with ER272 and/or the general PKC inhibitor Gö6850 at the indicated times. Data are the mean values ± S.E.M of nine independent measurements (*n* = 9). Statistical analysis: two tailed unpaired Student’s *t* test (****ER272 + Gö6850 vs. control at 30 min, *p* < 0.0001; *ER272 vs. control at 60 min, *p* = 0.0389; ***ER272 + Gö6850 vs. control at 60 min, *p* < 0.0001; *ER272 vs. control at 180 min, *p* = 0.0172; **ER272 + Gö6850 vs. control at 180 min, *p* < 0.0018).

In order to demonstrate the effect of ER272 on EGFR ligand release, we built a construct in which TGFα was expressed in a fusion protein flanked by GFP in the C-terminal domain and Cherry in the N-terminal (Fig. 5A). This construct was transfected into HEK293T cells and by using life imaging we quantified the variation in the proportion of mCherry/GFP in the cell membrane upon addition of ER272. Figure 5 shows that addition of vehicle to mCherry-TGFα-GFP transfected cultures of HEK293 did not induce any change in the mCherry/GFP ratio (movie 1). However, cells expressing the TGFα constructs produced a fast change in the mCherry/GFP ratio (movie 2) after ER272 addition, which was abolished by the presence of a classical (Gö6976) (Fig. 5B, C) or general (Gö6850) PKC inhibitor (Fig. 5B, C). To further ensure that the mCherry-labeled ligand was being released into the culture medium we analyzed fluorescence in the medium as a ratio to the green fluorescence in the cell fraction (Fig. 5D). Identical experiments were performed using a neuregulin 1 construct (Fig. 5E–G; movies 3, 4). The effect of ER272 on the red/green ratio when HEK293T cells were transfected with the neuregulin 1 construct was smaller than that found with TGFα and a smaller reduction of the red/green ratio was observed (Fig. 5E, F). Also, the amount of fluorescence found in the culture medium was smaller than that found with the TGFα construct (Fig. 5G).

## Discussion

We have found in here that intranasal administration of ER272 promotes proliferation of NPC in both neurogenic niches of the adult mouse brain. Remarkably, long-term intranasal administration of this small molecule facilitates differentiation of DG NSC toward a neuronal phenotype increasing the number of newborn neurons. Concomitantly, the treatment improves memory and learning tasks, having a particular impact on spatial memory. The effect on neurogenesis does not seem to impact cell cycle exit of quiescence, but it reduces astrogliogenesis. Finally, we have also elucidated the mechanisms of action of this compound, thus finding that ER272 stimulates neurogenesis by activating PKCα. ER272-induced classical PKC activation facilitates TGFα release and, to a lesser extent, neuregulin 1 release.

### Intranasal administration of ER272 promotes neurogenesis in the adult hippocampus and the SVZ

Previous reports have described the role of the diterpene with 12-deoxyphorobol structure ER272 in inducing proliferation of neural progenitors in vitro in cultures of NPC and in mice receiving single ICV injections of this molecule [20]. The study proposed this compound as a drug to induce neurogenesis. However, the study did not demonstrate whether this compound could exert an effect if administered using a non-invasive methodology or if longer treatments exerted a neurogenic effect within the niches improving brain tasks associated with neurogenesis such as memory and learning performance. We show that intranasal administration of 12-deoxyphorbol ER272 stimulates proliferation of progenitors in the SVZ after 7 days of treatment in agreement with the work of Geribaldi-Doldán et al. [20]. In this particular region, the number of ASCL^+^ transit amplifying undifferentiated progenitors [40, 41] is increased and this may be the reason for the enlarged number of DCX^+^ neuroblasts that have incorporated BrdU indicating a neurogenic role for this compound in the SVZ. On the contrary, no effect was observed in the generation of other cells types such as glial cells (GFAP^+^) in the SVZ. We also show that ER272 promotes the generation of neuroblasts in the DG (DCX^+^/BrdU^+^ cells) after a 7-day treatment, without affecting the number of cells that exit quiescence to enter cell cycle (Ascl^+^ cells) [42], indicating that this compound does not alter the preservation of the stem cell pool within this region while exerting its neurogenic effect. This effect was concomitant with a reduction in the number of GFAP^+^/BrdU^+^ cells. However, a long-term effect on gliogenesis was not observed. A preferential differentiation of hippocampal NSC into glial cells has been shown in the DG of mice models of ageing with a reduced neurogenesis [43, 44]. This suggested a possible effect of ER272 at preventing the astrogliogenesis that takes place in the aged brain or under neurodegeneration. However, in the young mice analyzed in here a long-term effect of the compound on glial cells has not been found.

Remarkably, we must highlight that a longer ER272 treatment (4-weeks)-induced neurogenesis in the DG increasing the number of newly generated neurons in the DG (NeuN^+^/BrdU^+^ cells) indicating that ER272 induces proliferation of cells that differentiate into neurons and survive within the niche for at least 2 weeks. Deficits in adult neurogenesis have been associated with cognitive impairments, and compromised hippocampal circuitry in mice models of neurodegenerative disease [45], suggesting again a possible role for this compound in improving cognitive performance in individuals with these pathologies.

### ER272 improves spatial memory and learning in adult healthy mice

Accordingly, the stimulation of neurogenesis in the DG was concomitant with an improvement in spatial memory and learning tasks of mice treated with ER272 for 28 days. This can be inferred from the improved performance of treated mice in the MWM test and in the “where” paradigm of the NOD test. In our hands, ER272 significantly improved “where” paradigm in the NOD test, in line with observations in the MWM for spatial memory. Whereas the implication of the hippocampus at this level remains under study, previous studies have reported the implication of the hippocampus for the object recognition memory [46] and that hippocampus is crucial for object location, object-in place and recency recognition memory [47]. The discrimination of simple geometric shapes, as those used in our NOD test, have been described to be hippocampal dependent [47]. The improvement of ER272 treated mice was similar to that found in healthy mice treated with insulin by intranasal administration [48]. This suggests that the effect of ER272 on memory tasks may be as potent as other neurogenic drugs such as insulin [48, 49]. However, we have not been able to directly demonstrate that the induction of neurogenesis is responsible for the improvement in memory and learning tasks. Notwithstanding, it has thoroughly been demonstrated that newborn neurons at different maturation stages may contribute to hippocampal circuits by either rewiring pre-existing circuits [50], or establishing new memory codes that determine pattern separation [51–53]. Therefore, it is likely that the enhanced neurogenesis found in mice treated with ER272 is responsible for the improvement in memory and learning tasks.

### ER272 mechanisms of action

ER272 effect on proliferation in vitro and in vivo is dependent on PKC activity in vitro [20]. However, the mechanism of action of ER272 needed to be elucidated. In here, we have determined that classical PKCα isozyme is responsible for the proliferative effect of ER272 in vitro. The role of PKCβ in the effect, was not as significant as that of PKCα. A similar diterpene with lathyrane structure, ELAC, with the capacity to induce proliferation of SVZ transit amplifying progenitors has previously been associated to the activation of classical PKCβ [26]. In light of these results it is likely that 12-deoxyphorbols have a higher affinity for the regulatory domain of PKCα whereas lathyrane molecules have a higher affinity for the regulatory domain of PKCβ.

Our results demonstrate the involvement of PKC in the neurogenic effect of ER272 in neurogenic niches in vivo since the PKC inhibitor abolished the neurogenic effect of ER272 in both the SVZ and DG. In addition, our in vitro findings also assign a role for PKCα in neurogenesis in both niches participating in the release of TGFα and less significantly neuregulin. The effect of ER272 on TGFα release in vivo, is supported by the fact that ICV injections of ER272 promote proliferation of EGFR^+^ undifferentiated progenitors in the SVZ.

Classical and novel PKC isozymes can be activated by nonphysiological molecules such as phorbol esters. These tetracyclic diterpenoids activate PKC because they mimic the action of diacylglycerol [54]. Recent reports demonstrate that activation of specific PKC isozymes determines ADAM17 selectivity for its different substrates. Thus, TGFα phosphorylation by classical PKCα activated by PMA facilitates its shedding mediated by ADAM17 [17, 18]. On the contrary, activation of novel PKCδ is required for ADAM17 mediated secretion of neuregulin. Phosphorylation of serine 286 in the cytoplasmic domain of neuregulin catalyzed by PKCδ facilitates the scission of its ectodomain [17, 18] releasing neuregulin into the extracellular medium. Overall, this indicates that ADAM17 substrate specificity and selectivity is mediated by the activation of different PKC isozymes, thus playing a key role in the secretion of different types of ligands [17, 18]. We show that treatment with ER272 of HEK293T cells transfected with a TGFα flanked by mCherry and GFP induces the release of TGFα quantified as the ratio of mCherry fluorescence released outside the cells. In addition, a smaller release of neuregulin 1 has also been observed in cells transfected with a similar construct in which neuregulin 1 is fused to mCherry and GFP in a similar way. These results indicate that ER272 exerts a more potent effect at releasing TGFα than other ligands such as neuregulin. It is likely that ER272 has a higher affinity for the regulatory site of classical PKC isozymes, responsible for the release of TGFα, than for that of novel isozymes, responsible for the release of neuregulin.

An effect of neuregulin in DG neurogenesis and behavior has previously been reported by Mahar et al. who find that neuregulin 1 administration increases ErbB3 phosphorylation in the DG, together with a neurogenic effect of neuregulin 1 on the generation of newborn neurons [14]. Neuregulin 1 and ErbB3 downregulation in granule cells neurons within the hippocampus has been reported in suicidal subjects, an effect that is reverted with antidepressants suggesting a role for neuregulin 1 and antidepressants in the prevention of this particular behavior [13]. Therefore, treatment with ER272 might induce the release of neuregulin 1, thus acting as a putative compound in the development of antidepressant drugs. Also, neuregulin mutations have been involved in cognitive impairment in schizophrenic [55] and other neuregulins such as neuregulin 2 have been shown to contribute to the maturation of granule cell neurons, thus indicating a role for neuregulins in the regulation of cognitive tasks. An effect of TGFα on hippocampal neurogenesis and memory performance has also been described in the hippocampus in models of ischemia [15]. TGFα expression increases as a response to injuries, however, a physiological role for TGFα on hippocampal neurogenesis, still remains to be investigated. In here, we show that ER272 in vitro mimics the effect of EGFR ligands mediating its effect through pathways initiated by ligands such as TGFα (MAPK and PI3K-AKT signaling pathways) thus supporting the hypothesis of its role in TGFα release. Interestingly, experimental models in which these pathways are inhibited as a consequence of metabolic alterations such as mice models of hyperhomocysteinemia or the mice model of hypermethioninemia Gnmt−/− mice show a deficient neurogenesis in both the DG and the SVZ [28, 29, 37] and a concomitant cognitive impairment [37].

In conclusion, we have found that intranasal treatment of mice with ER272 promotes neurogenesis and improves cognitive performance. This compound facilitates the release of growth factors that activate ErbB receptors such as neuregulin or TGFα, suggesting a role for ER272 as a possible drug to promote neurogenesis in diseases associated with cognitive impairment.

## Funding and disclosure

This work was supported by the Spanish Ministerio de Ciencia, Innovación y Universidades (grant numbers RTI-2018-099908-B-C21 granted to CC and RTI-2018-099908-B-C22 granted to RGH), and MICINN/FEDER granted to CC and BFU2016-75038R granted to MGA) and Consejería de Economía, Conocimiento, Empresas y Universidades (grant number FEDER-UCA18-106647). Authors claim no competing interests.

## Supplementary information

Supplementary methods and antibody tables

Supplemmentary Figures 1–8

Supplementary movie 1

Supplementary movie 2

Supplementary movie 3

Supplementary movie 4

## References

[1] Llorens-Bobadilla E, Zhao S, Baser A, Saiz-Castro G, Zwadlo K, Martin-Villalba A (2015). Single-Cell Transcriptomics Reveals a Population of Dormant Neural Stem Cells that Become Activated upon Brain Injury. Cell Stem Cell.

[2] Goldman S (2003). Glia as neural progenitor cells. Trends Neurosci.

[3] Alvarez-Buylla A, Garcia-Verdugo JM (2002). Neurogenesis in adult subventricular zone. J Neurosci.

[4] Gage FH, Ray J, Fisher LJ (1995). Isolation, characterization, and use of stem cells from the CNS. Annu Rev Neurosci.

[5] Doetsch F, Garcia-Verdugo JM, Alvarez-Buylla A (1997). Cellular composition and three-dimensional organization of the subventricular germinal zone in the adult mammalian brain. J Neurosci.

[6] Aimone JB, Deng W, Gage FH (2011). Resolving new memories: a critical look at the dentate gyrus, adult neurogenesis, and pattern separation. Neuron..

[7] Deng W, Aimone JB, Gage FH (2010). New neurons and new memories: how does adult hippocampal neurogenesis affect learning and memory?. Nat Rev Neurosci.

[8] Snyder JS, Soumier A, Brewer M, Pickel J, Cameron HA (2011). Adult hippocampal neurogenesis buffers stress responses and depressive behaviour. Nature..

[9] Snyder JS, Cameron HA (2012). Could adult hippocampal neurogenesis be relevant for human behavior?. Behav Brain Res.

[10] Boldrini M, Butt TH, Santiago AN, Tamir H, Dwork AJ, Rosoklija GB (2014). Benzodiazepines and the potential trophic effect of antidepressants on dentate gyrus cells in mood disorders. Int J Neuropsychopharmacol.

[11] Boldrini M, Santiago AN, Hen R, Dwork AJ, Rosoklija GB, Tamir H (2013). Hippocampal granule neuron number and dentate gyrus volume in antidepressant-treated and untreated major depression. Neuropsychopharmacology..

[12] Malberg JE, Eisch AJ, Nestler EJ, Duman RS (2000). Chronic antidepressant treatment increases neurogenesis in adult rat hippocampus. J Neurosci.

[13] Mahar I, Labonte B, Yogendran S, Isingrini E, Perret L, Davoli MA (2017). Disrupted hippocampal neuregulin-1/ErbB3 signaling and dentate gyrus granule cell alterations in suicide. Transl Psychiatry.

[14] Mahar I, MacIsaac A, Kim JJ, Qiang C, Davoli MA, Turecki G (2016). Effects of neuregulin-1 administration on neurogenesis in the adult mouse hippocampus, and characterization of immature neurons along the septotemporal axis. Sci Rep..

[15] Alipanahzadeh H, Soleimani M, Soleimani Asl S, Pourheydar B, Nikkhah A, Mehdizadeh M (2014). Transforming Growth Factor-alpha Improves Memory Impairment and Neurogenesis Following Ischemia Reperfusion. Cell J..

[16] Blobel CP (2005). ADAMs: key components in EGFR signalling and development. Nat Rev.

[17] Dang M, Armbruster N, Miller MA, Cermeno E, Hartmann M, Bell GW (2013). Regulated ADAM17-dependent EGF family ligand release by substrate-selecting signaling pathways. Proc Natl Acad Sci USA.

[18] Dang M, Dubbin K, D’Aiello A, Hartmann M, Lodish H, Herrlich A (2011). Epidermal growth factor (EGF) ligand release by substrate-specific a disintegrin and metalloproteases (ADAMs) involves different protein kinase C (PKC) isoenzymes depending on the stimulus. J Biol Chem.

[19] Geribaldi-Doldan N, Gomez-Oliva R, Dominguez-Garcia S, Nunez-Abades P, Castro C (2019). Protein Kinase C: targets to Regenerate Brain Injuries?. Front Cell Dev Biol.

[20] Geribaldi-Doldan N, Flores-Giubi E, Murillo-Carretero M, Garcia-Bernal F, Carrasco M, Macias-Sanchez AJ (2015). 12-Deoxyphorbols Promote Adult Neurogenesis by Inducing Neural Progenitor Cell Proliferation via PKC Activation. Int J Neuropsychopharmacol.

[21] Kilkenny C, Browne W, Cuthill IC, Emerson M, Altman DG, Group NCRRGW. (2010). Animal research: reporting in vivo experiments: the ARRIVE guidelines. Br J Pharm.

[22] McGrath JC, Drummond GB, McLachlan EM, Kilkenny C, Wainwright CL (2010). Guidelines for reporting experiments involving animals: the ARRIVE guidelines. Br J Pharm.

[23] Thorne RG, Pronk GJ, Padmanabhan V, Frey WH (2004). Delivery of insulin-like growth factor-I to the rat brain and spinal cord along olfactory and trigeminal pathways following intranasal administration. Neuroscience..

[24] Francis GJ, Martinez JA, Liu WQ, Xu K, Ayer A, Fine J (2008). Intranasal insulin prevents cognitive decline, cerebral atrophy and white matter changes in murine type I diabetic encephalopathy. Brain..

[25] Marks DR, Tucker K, Cavallin MA, Mast TG, Fadool DA (2009). Awake intranasal insulin delivery modifies protein complexes and alters memory, anxiety, and olfactory behaviors. J Neurosci.

[26] Murillo-Carretero M, Geribaldi-Doldan N, Flores-Giubi E, Garcia-Bernal F, Navarro-Quiroz EA, Carrasco M (2017). ELAC (3,12-di-O-acetyl-8-O-tigloilingol), a plant-derived lathyrane diterpene, induces subventricular zone neural progenitor cell proliferation through PKCbeta activation. Br J Pharm.

[27] Garcia-Bernal F, Geribaldi-Doldan N, Dominguez-Garcia S, Carrasco M, Murillo-Carretero M, Delgado-Ariza A (2018). Protein Kinase C Inhibition Mediates Neuroblast Enrichment in Mechanical Brain Injuries. Front Cell Neurosci.

[28] Rabaneda LG, Carrasco M, Lopez-Toledano MA, Murillo-Carretero M, Ruiz FA, Estrada C (2008). Homocysteine inhibits proliferation of neuronal precursors in the mouse adult brain by impairing the basic fibroblast growth factor signaling cascade and reducing extracellular regulated kinase 1/2-dependent cyclin E expression. FASEB J.

[29] Rabaneda LG, Geribaldi-Doldan N, Murillo-Carretero M, Carrasco M, Martinez-Salas JM, Verastegui C (2016). Altered regulation of the Spry2/Dyrk1A/PP2A triad by homocysteine impairs neural progenitor cell proliferation. Biochim Biophys Acta.

[30] Henley R, Chandrasekaran V, Giulivi C (2019). Computing neurite outgrowth and arborization in superior cervical ganglion neurons. Brain Res Bull.

[31] Geribaldi-Doldan N, Carrasco M, Murillo-Carretero M, Dominguez-Garcia S, Garcia-Cozar FJ, Munoz-Miranda JP (2018). Specific inhibition of ADAM17/TACE promotes neurogenesis in the injured motor cortex. Cell Death Dis.

[32] Ramos-Rodriguez JJ, Ortiz O, Jimenez-Palomares M, Kay KR, Berrocoso E, Murillo-Carretero MI (2013). Differential central pathology and cognitive impairment in pre-diabetic and diabetic mice. Psychoneuroendocrinology..

[33] Dere E, Huston JP, De Souza Silva MA (2005). Episodic-like memory in mice: simultaneous assessment of object, place and temporal order memory. Brain Res Brain Res Protoc.

[34] Infante-Garcia C, Ramos-Rodriguez JJ, Hierro-Bujalance C, Ortegon E, Pickett E, Jackson R (2018). Antidiabetic Polypill Improves Central Pathology and Cognitive Impairment in a Mixed Model of Alzheimer’s Disease and Type 2 Diabetes. Mol Neurobiol.

[35] Segado-Arenas A, Infante-Garcia C, Benavente-Fernandez I, Sanchez-Sotano D, Ramos-Rodriguez JJ, Alonso-Ojembarrena A (2018). Cognitive Impairment and Brain and Peripheral Alterations in a Murine Model of Intraventricular Hemorrhage in the Preterm Newborn. Mol Neurobiol.

[36] Curtis MJ, Abernethy DR (2015). Revision of instructions to authors for pharmacology research and perspectives: enhancing the quality and transparency of published work. Pharmacol Res Perspect.

[37] Carrasco M, Rabaneda LG, Murillo-Carretero M, Ortega-Martinez S, Martinez-Chantar ML, Woodhoo A (2014). Glycine N-methyltransferase expression in the hippocampus and its role in neurogenesis and cognitive performance. Hippocampus..

[38] Nunez-Abades PA, He F, Barrionuevo G, Cameron WE (1994). Morphology of developing rat genioglossal motoneurons studied in vitro: changes in length, branching pattern, and spatial distribution of dendrites. J Comp Neurol.

[39] Carrascal L, Nieto-Gonzalez JL, Torres B, Nunez-Abades P (2009). Changes in somatodendritic morphometry of rat oculomotor nucleus motoneurons during postnatal development. J Comp Neurol.

[40] Ponti G, Obernier K, Guinto C, Jose L, Bonfanti L, Alvarez-Buylla A (2013). Cell cycle and lineage progression of neural progenitors in the ventricular-subventricular zones of adult mice. Proc Natl Acad Sci USA.

[41] Parras CM, Galli R, Britz O, Soares S, Galichet C, Battiste J (2004). Mash1 specifies neurons and oligodendrocytes in the postnatal brain. EMBO J.

[42] Castro DS, Martynoga B, Parras C, Ramesh V, Pacary E, Johnston C (2011). A novel function of the proneural factor Ascl1 in progenitor proliferation identified by genome-wide characterization of its targets. Genes Dev.

[43] Diaz-Moreno M, Armenteros T, Gradari S, Hortiguela R, Garcia-Corzo L, Fontan-Lozano A (2018). Noggin rescues age-related stem cell loss in the brain of senescent mice with neurodegenerative pathology. Proc Natl Acad Sci USA.

[44] Encinas JM, Michurina TV, Peunova N, Park JH, Tordo J, Peterson DA (2011). Division-coupled astrocytic differentiation and age-related depletion of neural stem cells in the adult hippocampus. Cell Stem Cell.

[45] Hollands C, Tobin MK, Hsu M, Musaraca K, Yu TS, Mishra R (2017). Depletion of adult neurogenesis exacerbates cognitive deficits in Alzheimer’s disease by compromising hippocampal inhibition. Mol Neurodegener.

[46] Broadbent NJ, Gaskin S, Squire LR, Clark RE (2010). Object recognition memory and the rodent hippocampus. Learn Mem.

[47] Grether GF (2011). The neuroecology of competitor recognition. Integr Comp Biol.

[48] Ramos-Rodriguez JJ, Sanchez-Sotano D, Doblas-Marquez A, Infante-Garcia C, Lubian-Lopez S, Garcia-Alloza M (2017). Intranasal insulin reverts central pathology and cognitive impairment in diabetic mother offspring. Mol Neurodegener.

[49] Mao YF, Guo Z, Zheng T, Jiang Y, Yan Y, Yin X (2016). Intranasal insulin alleviates cognitive deficits and amyloid pathology in young adult APPswe/PS1dE9 mice. Aging Cell.

[50] Goodwin SJ (2018). Neurogenesis: remembering all or forgetting some. J Neurophysiol.

[51] McAvoy K, Besnard A, Sahay A (2015). Adult hippocampal neurogenesis and pattern separation in DG: a role for feedback inhibition in modulating sparseness to govern population-based coding. Front Syst Neurosci.

[52] Toda T, Parylak SL, Linker SB, Gage FH (2019). The role of adult hippocampal neurogenesis in brain health and disease. Mol Psychiatry.

[53] Toda T, Gage FH (2018). Review: adult neurogenesis contributes to hippocampal plasticity. Cell Tissue Res.

[54] Newton AC (1995). Protein Kinase C: seeing two domains. Curr Biol.

[55] Cho Y, Ryu S, Huh I, Cho EY, Oh H, Lee YS (2015). Effects of genetic variations in NRG1 on cognitive domains in patients with schizophrenia and healthy individuals. Psychiatr Genet.

