# Supplementary material for: Effects of classical PKC activation on hippocampal neurogenesis and cognitive performance: mechanism of action
Source: Neuropsychopharmacology. 2020 Dec 17;46(6):1207–19. doi: 10.1038/s41386-020-00934-y (PMC8115324; doi:10.1038/s41386-020-00934-y)
Supplement: Supplementary file 1 — Supplementary methods and antibody tables [file 41386_2020_934_MOESM1_ESM.pdf]

Supplementary methods

(enlarged version of the methodology)

Reagents

Isolation and purification of the 12-deoxyphorbol ER272 (CAS:25090-74-8) was performed in our laboratory as previously described [1]. In brief, compound was obtained by isolation and purification from methanolic extracts of the latex from *E. resinífera*. The methanolic fraction was subjected to silica gel column chromatography with an increasing gradient of ethyl acetate in hexane as mobile phase and monitored by TLC. Further semi-preparative and analytical HPLC yielded compounds with spectroscopic (1H and 13C NMR) and spectrometric (HRMS) data consistent with previously described 13-*O*-isobutiroyl-12-deoxiphorbol (ER272) [2]. PKC inhibitors were purchased from Sigma-Aldrich (St. Louis, MO) and Calbiochem (Millipore, Billerica, MA). The general inhibitor of PKC bisindolylmaleimide I (Gö6850) and the classical PKC inhibitor (Gö6976) were added to cell cultures at final concentrations of 5µM and 1 µM, respectively and were added 30 minutes before the addition of the PKC activators. Other products, unless otherwise indicated, were purchased from Sigma-Aldrich (St. Louis, MO, USA).

Animals

SVZ derived neural progenitor cells (NPC) were isolated from 7-day postnatal CD1 mice and used for *in vitro* experiments. For *in vivo* experiments 2-month-old male CD1 mice were used. Animals were housed under controlled conditions of temperature (21-23°C) and light (LD 12:12) with free access to food (AO4 standard maintenance diet, SAFE, Épinay-sur-Orge, France) and water. Care and handling of animals were performed according to the Guidelines of the European Union Council (2010/63/EU), and the Spanish regulations (65/2012 and RD53/2013) for the use of laboratory animals. All studies involving animals are reported in accordance with the ARRIVE guidelines for reporting experiments involving animals [3,4]

Intranasal administration of ER272

ER272, was delivered intranasally as previously described [5-7]; in brief, treatments were administered manually while the animal was placed in a standing position with an extended neck as previously described [7]. 18 µL of each solution (5 µM ER272 in saline, or saline as vehicle) was delivered over both nasal cavities alternating 3µL/each using a micropipette. Mouse was maintained in such position for 10 additional seconds to ensure all fluid was inhaled. In all experiments, mice were coded, treatment (vehicle or ER272) was assigned randomly to code numbers and applied. In addition, blind quantifications were performed to avoid subjective biases.

ICV administration of ER272

Adult mice were anesthetized and placed on a stereotaxic frame to administrate ER272 (1 µM) or only vehicle as previously described [1,8]. In brief, 2 months old adult male mice were anaesthetized with ketamine/xylazine

and placed on a stereotaxic frame (Kopf Instruments). We made a small trepanation 0.8mm lateral to Bregma, to introduce the needle of a 5-µL Hamilton syringe 2.4mm below the brain surface. A solution of ultra-filtered ER272, in PBS (1 µM final concentration) (n= 6), or vehicle (n=6), were injected ipsilaterally into the right lateral ventricles. 2 µL single injections were give per brain through a time-frame of 10 minutes; afterwards, the Hamilton syringe was left another 5 minutes before removal. Animals received daily injections of bromodeoxyuridine (BrdU) (120mg/kg), during 3 days starting the day of the surgical procedure. Mice were then deeply anaesthetized with pentobarbital and perfused with 4% PFA via ascending aorta. Brains were removed and sliced in 30-µm serial sections from Bregma 2.1 to -3.8, where the SVZ and hippocampus are contained.

Brain processing and immunohistochemistry

At the end of the treatment mice were then deeply anaesthetized with pentobarbital and perfused with 4% PFA via ascending aorta; brains were then removed and–sliced using a cryotome into 30 µm sections. Immunohistochemistry was performed as previously described [1,8,9]. See antibodies in supplementary tables 1 and 2.

Quantification of neurogenesis in brain sections

Cells positive for BrdU, DCX, GFAP, EGFR, nestin, or Ascl1 in the SVZ and DG were estimated as described [10,11]. Positive cells were counted throughout the entire DG area or lateral and laterodorsal walls of the lateral ventricles in every fifth section; 14-16 sections per brain where analyzed under fluorescence microscopy at 20X magnification. Mice were coded depending on the treatment and quantification of cells in brain slices was done in blinded analysis. Cells in the SVZ and DG of both brain hemispheres have been quantified unless otherwise indicated.

SVZ-derived NPC isolation and culture

SVZ- derived cells were isolate following the same procedure described in Rabaneda et al, 2008 [10]. NPCs were obtained from the SVZ of 7-day postnatal mice following the procedure previously described [10] . Neurosphere cultures were maintained in defined medium (DM) composed of Dulbecco's modified Eagle's medium/F12 medium (1:1vol/vol) plus 1mg/L gentamicin (GIBCO) and the B27 supplement (Invitrogen, Carlsbad, CA). EGF (20ng/mL) and bFGF (10ng/mL; both from PeproTech, Frankfurt, Germany) were added to DM for culture expansion. Growth factors EGF and bFGF were used as indicated in the figure legends

Neurosphere assay

To test the effects of ER272 on NPC proliferation, single cells from mechanically disaggregated neurospheres were plated onto anti-adherent 96-well plates at a density of 20,000 cells/mL in defined medium + 10ng/mL bFGF, 20 ng/mL of EGF or both as indicated in the figure legends. ER272 and other pharmacological agents were added at the time of seeding, and all conditions were run in triplicates. Neurosphere number and size were

measured as previously described [11]. Inhibitors were added 30 minutes before the addition of ER272. Results were obtained from a minimum of 3 independent experiments performed with triplicate samples.

Neurosphere treatment and transfection

Neurosphere cells were transfected with specific siRNA SmartPool One target siRNA from Horizon (Cambridge, UK) against each specific PKC isozyme using Lipofectamine 2000, following the manufacturer's instructions as previously described [8]. ER272 was added and cells were maintained for 48 additional hours before being used for flow cytometry.

Flow cytometry

For proliferation studies, cells were disaggregated from the neurospheres and fixed in 4% PFA rinsed with PBS and centrifuged (300 x *g*; 5 min). Cells were incubated in a blocking solution (1 $mg \cdot mL^{-1}$ bovine serum albumin and 0.3% Triton X100) followed by an incubation with fluorescent antibody (supplementary table S1). Cells were then rinsed in PBS, resuspended in FACS buffer and analyzed in a Attune NxT flow cytometer (Invitrogen).

Cloning of human Neuregulin and TGFα cDNA fused to GFP and Cherry

Full-length cDNA encoding the membrane-bound isoform of human pro-neuregulin-1 β1-type (NRG1, NCBI reference sequence: NP_039250.2) with mCherry cDNA inserted between nucleotides 93 and 94 of NRG1 open reading frame was cloned into pEGFP-N1 to add EGFP cDNA to the 3′ end. Construct was synthesized by GeneCust (Boynes, France) to generate the mCherry-NRG1-GFP construct. The mCherry-TGFα-GFP construct containing the human transforming growth factor alpha (TGFA, NCBI reference sequence: NM_003236.4), containing mCherry cDNA between nucleotides 126 and 127 of TGFA was built using the same strategy and synthesized by GeneCust (Boynes, France).

HEK293T culture and transfection

HEK293T obtained from ATCC (Manassas, VA, USA) were cultured and transfected as previously described [12]. HEK293T cells were obtained from ATCC (Manassas, VA, USA). They were cultured in DMEM at 37 °C and 5% CO2 (Thermo Fisher Scientific, Inc., Rockford, IL, USA), supplemented with fetal bovine serum (10%), 1× GlutaMA^{TM}-I (Thermo Fisher Scientific, Inc., Rockford, IL, USA) and penicillin/streptomycin (1%). Cells were passaged, seeded and allowed to attach for 24 h. Lipofectamine 2000 (Invitrogen; Carlsbad, CA, USA) was used for transfection of these plasmids. Afterwards, medium was changed to eliminate Lipofectamine. After an overnight incubation, the cells were starved for at least 30 minutes in serum-free Fluorobrite DMEM (Thermo Fisher Scientific) containing 1% P/S, 0.25% bovine serum albumin (BSA), and 1× GlutaMAX^{TM}-I. and cells were used either in time-lapse or fluorescence experiments.

Time lapse experiments and fluorescence analysis of mCherry-fused TGFα or neuregulin in the culture medium of HEK293T

Transfected HEK293T cells were plated in 35 mm high µ–dishes (Ibidi, Munich, Germany). Cells were treated with EOF2 or inhibitors, as described in the results and figure legends. Time-lapse assays were performed with a Zeiss Axio Observer.Z1-Inverted Microscope, using a plan-apochromat 40x/0.95 Korr M27 air objective lens. Images of transfected cells were obtained every 1 min. Captured images were processed using ZEN lite software and the efficiency of NRG1 and TGFα cleavage determined by analyzing the mCherry/GFP fluorescence intensity over the entire cell areas. The mCherry/GFP ratios were calculated and normalized to the average ratio measured before stimulation with ER272 using the Microsoft Excel software. Ratiometric images were built using ImageJ software, after background subtraction, the mCherry/GFP image was calculated dividing mCherry channel by the GFP channel. For each pixel, a pseudocolor scale is used for coding the ratio.

Morris water maze (MWM)

Spatial memory and learning tasks were analyze starting 10 days before sacrifice using the MWM test in control and treated mice as previously described [13] and shown in figure legend. Briefly, the maze consisted of a circular tank of water (Ø = 0.95 m). Four equal virtual quadrants were indicated by geometric cues mounted on the walls. The hidden escape platform was located 2–3 cm below the white-coloured water surface. Water temperature was 21 ± 1 °C. A camera attached to a computer and Smart software (Panlab, Spain) was located above the maze. The testing consisted in an acquisition phase followed by a retention phase. Acquisition consisted of four trials per day during 4 days. During this phase the platform, located in quadrant 2 was submerged. Time was limited to 60 s/trial with intervals of 10 min between trials. If the animal did not find the platform, it was placed on it for 10 s. Two retention tests were performed 24 h (retention 1) and 72 h (retention 2) after finishing the acquisition phase. During these tests, the platform was removed and mice were allowed to swim for 60s. The time spent in the quadrant where the platform was previously located (quadrant 2) was recorded using SMART system (Panlab, Spain). Swimming speed was also quantified to detect any motor activity dysfunction that could bias the learning and memory assessment.

Motor activity and new object discrimination (NOD) task

Motor activity was analyzed measuring the distance travelled by each mice during a 30 s period before initiating the NOD test. Then integrated episodic memory for the paradigms “what”, “when” and “where” was analyzed as described in previous reports [13]. One day after finishing the MWM task, locomotor activity was analyzed in all animal gropus. We measured the distance travelled by the mice for 30 min in a transparent rectangular box (22-cm long × 44-cm width × 40-cm high) using SMART system (Panlab, Spain). In addition, in order to assess any anxiety-like behavior, distance travelled was analyzed in the proximity of the walls, as well as in the center of the boxes (10 cm from the border). One day after actimetry, mice continued with the NOD test to

analyze episodic memory. The second day, mice were exposed to two objects, for habituation purposes, not used again during the object exploration task on day 3. On day 3 each mouse received two sample trials and a test trial. On the first sample trial, mice were placed into the center of the box containing three copies of a novel object (blue balls) arranged in a triangle-shaped spatial configuration and allowed to explore them for 5 min. After a delay of 30 min, the mice received a second sample trial with four novel objects (red cones), arranged in a quadratic-shaped spatial configuration, for 5 min. After a delay of 30 min, the mice received a test trial with two copies of the object from sample trial 2 (recent objects) placed in the same position and two copies of the object from sample trial 1 (familiar objects): one of them in the same position (familiar non-displaced object) and the other one in a new position (familiar displaced object). Integrated episodic memory for "what," "where," and "when" was analyzed: "What" was defined as the difference in time exploring familiar and recent objects, "where" was defined as the difference in time exploring displaced and non-displaced objects, and "when" was defined as the difference between time exploring familiar non-displaced and recent non-displaced objects. Motor function was also analyzed in the rotarod (Panlab, Spain). Briefly, mice were placed in the rotarod facing away the experimenter. The rod accelerated from 0 to 30 rpm over 3 min and final revolutions per minute for each mice were recorded.

Statistical analysis

The data and statistical analysis comply with the recommendations on experimental design and analysis in pharmacology [14]. Statistical analysis was performed using the computer program IBM SPSS Statistics 22. Unless otherwise indicated, normal distribution of the data was first analyzed using a Shapiro-Wilks test. Then, a Brown Forsythe test was performed to test the equality of variances. Afterwards, when more than one treatment group were compared, statistical analyses were performed using one-way ANOVA followed by a post-hoc Bonferroni's test unless otherwise indicated. Two way-ANOVA (group x day) was used in the que acquisition phase of the MWM. A Student's *t* test was used when only one treatment group was compared with the control. Differences were considered significant at values of $p < 0.05$. In general, sample size used in statistical analysis were n=6-10 for *in vivo* experiments and n = 5-9 for *in vitro* experiments. Sample sizes were chosen based on previous works related to this one [8,11,12,15].

1 Geribaldi-Doldan N, Flores-Giubi E, Murillo-Carretero M, Garcia-Bernal F, Carrasco M, Macias-Sanchez AJ, et al. 12-Deoxyphorbols Promote Adult Neurogenesis by Inducing Neural Progenitor Cell Proliferation via PKC Activation. Int J Neuropsychopharmacol. 2015;19(1).
2 Schmidt RJ, Evans FJ. The succulent Euphorbias of Nigeria. II. aliphatic diterpene esters of the latices of E. poisonii Pax. and E. unispina N.E. Br. Lloydia. 1977;40(3):225-9.
3 Kilkenny C, Browne W, Cuthill IC, Emerson M, Altman DG, Group NCRRGW. Animal research: reporting in vivo experiments: the ARRIVE guidelines. Br J Pharmacol. 2010;160(7):1577-9.
4 McGrath JC, Drummond GB, McLachlan EM, Kilkenny C, Wainwright CL. Guidelines for reporting experiments involving animals: the ARRIVE guidelines. Br J Pharmacol. 2010;160(7):1573-6.
5 Thorne RG, Pronk GJ, Padmanabhan V, Frey WH, 2nd. Delivery of insulin-like growth factor-I to the rat brain and spinal cord along olfactory and trigeminal pathways following intranasal administration. Neuroscience. 2004;127(2):481-96.
6 Francis GJ, Martinez JA, Liu WQ, Xu K, Ayer A, Fine J, et al. Intranasal insulin prevents cognitive decline, cerebral atrophy and white matter changes in murine type I diabetic encephalopathy. Brain. 2008;131(Pt 12):3311-34.
7 Marks DR, Tucker K, Cavallin MA, Mast TG, Fadool DA. Awake intranasal insulin delivery modifies protein complexes and alters memory, anxiety, and olfactory behaviors. J Neurosci. 2009;29(20):6734-51.
8 Murillo-Carretero M, Geribaldi-Doldan N, Flores-Giubi E, Garcia-Bernal F, Navarro-Quiroz EA, Carrasco M, et al. ELAC (3,12-di-O-acetyl-8-O-tigloilingol), a plant-derived lathyrane diterpene, induces subventricular zone neural progenitor cell proliferation through PKCbeta activation. Br J Pharmacol. 2017;174(14):2373-92.
9 Garcia-Bernal F, Geribaldi-Doldan N, Dominguez-Garcia S, Carrasco M, Murillo-Carretero M, Delgado-Ariza A, et al. Protein Kinase C Inhibition Mediates Neuroblast Enrichment in Mechanical Brain Injuries. Front Cell Neurosci. 2018;12:462.
10 Rabaneda LG, Carrasco M, Lopez-Toledano MA, Murillo-Carretero M, Ruiz FA, Estrada C, et al. Homocysteine inhibits proliferation of neuronal precursors in the mouse adult brain by impairing the basic fibroblast growth factor signaling cascade and reducing extracellular regulated kinase 1/2-dependent cyclin E expression. FASEB J. 2008;22(11):3823-35.
11 Rabaneda LG, Geribaldi-Doldan N, Murillo-Carretero M, Carrasco M, Martinez-Salas JM, Verastegui C, et al. Altered regulation of the Spry2/Dyrk1A/PP2A triad by homocysteine impairs neural progenitor cell proliferation. Biochim Biophys Acta. 2016;1863(12):3015-26.
12 Geribaldi-Doldan N, Carrasco M, Murillo-Carretero M, Dominguez-Garcia S, Garcia-Cozar FJ, Munoz-Miranda JP, et al. Specific inhibition of ADAM17/TACE promotes neurogenesis in the injured motor cortex. Cell Death Dis. 2018;9(9):862.
13 Ramos-Rodriguez JJ, Ortiz O, Jimenez-Palomares M, Kay KR, Berrocoso E, Murillo-Carretero MI, et al. Differential central pathology and cognitive impairment in pre-diabetic and diabetic mice. Psychoneuroendocrinology. 2013;38(11):2462-75.
14 Curtis MJ, Abernethy DR. Revision of instructions to authors for pharmacology research and perspectives: enhancing the quality and transparency of published work. Pharmacol Res Perspect. 2015;3(2).
15 Carrasco M, Rabaneda LG, Murillo-Carretero M, Ortega-Martinez S, Martinez-Chantar ML, Woodhoo A, et al. Glycine N-methyltransferase expression in the hippocampus and its role in neurogenesis and cognitive performance. Hippocampus. 2014;24(7):840-52.

Primary and Secondary Antibody Tables

Antibody	Host	Isotype	Dilution	Epitope retrieval	Staining pattern	Source	Reference
Anti-BrdU	mouse	monoclonal	1:100	BrdU, proliferation marker	nuclear	Dako (Hamburg, Germany)	Z0334
Anti-BrdU	rat	monoclonal	1:200	BrdU, proliferation marker	nuclear	Abcam (Cambridge, UK)	AB6326
Anti-ASCL1	mouse	monoclonal	1:50	ASCL1	nuclear	BD Pharmigen (San Jose, CA, USA)	556604
Anti-DCX	rabbit	polyclonal	1:500	DCX, neuroblast marker	cytoplasmic	Abcam (Cambridge, UK).	ab6142
Anti-EGFR	sheep	polyclonal	1:200	EGFR, epidermic growth factor receptor	cytoplasmic	Merk Millipore (Billerica, Ma, USA)	06-847
Anti-GFAP	mouse	polyclonal	1:200	GFAP, glial marker	cytoplasmatic	Cell Signaling (Beverly, MA, USA)	3670
Anti-GFAP	rabbit	polyclonal	1:3000	GFAP, glial marker	cytoplasmatic	Dako (Hamburg, Germany)	Z0334
Anti-NeuN	mouse	monoclonal	1:500	NeuN, mature neurons	nuclear	Abcam (Cambridge, UK)	ab104225
Anti-β-III-tubulin	mouse	monoclonal	1:1000	β-III-tubulin, neuronal marker	cytoplasmic	StemCell Technology (Boston, MA, USA)	60052
Alexa Fluor 700 Anti-Ki67	Mouse, clone B56 (ROU)	monoclonal	1:200	Ki67, proliferation marker	nuclear	BD Bioscience	561277
Anti-S100β	rabbit	polyclonal	1:500	S-100β, astrocyte marker	cytoplasmic	Abcam (Cambridge, UK)	ab41548

Supplementary table 1 (Primary antibodies): List of primary antibodies used in the study. Specifying host, isotype, dilution used, epitope retrieval, staining pattern, source and reference.

Antibody	Host	Dilution	Fluorescence	Source	Reference
Alexa Flour-anti-mouse	donkey	1:1000	488	Invitrogen (Carlsbad, CA, USA)	A-21206
Alexa Flour-anti-mouse	donkey	1:1000	594	Invitrogen (Carlsbad, CA, USA)	A-21203
Alexa Flour-anti-mouse	goat	1:1000	405	Invitrogen (Carlsbad, CA, USA)	A-31553
Alexa Flour-anti-rat	donkey	1:1000	594	Invitrogen (Carlsbad, CA, USA)	A-11007
Alexa Flour-anti-rat	donkey	1:1000	488	Invitrogen (Carlsbad, CA, USA)	A-21208
Alexa Flour-anti-goat	donkey	1:1000	594	Invitrogen (Carlsbad, CA, USA)	A-11058
Alexa Flour-anti-mouse	goat	1:1000	546	Invitrogen (Carlsbad, CA, USA)	A-21123
Alexa Fluor-anti-rabbit	donkey	1:1000	594	Invitrogen (Carlsbad, CA, USA)	A-21207

Supplementary table 2 (Secondary antibodies): List of secondary antibodies used in the study. Specifying host, dilution used, fluorescence conjugated, source and reference.
