# Supplementary material for: Effects of classical PKC activation on hippocampal neurogenesis and cognitive performance: mechanism of action
Source: Neuropsychopharmacology. 2020 Dec 17;46(6):1207–19. doi: 10.1038/s41386-020-00934-y (PMC8115324; doi:10.1038/s41386-020-00934-y)
Supplement: Supplementary file 2 — Supplemmentary Figures 1–8 [file 41386_2020_934_MOESM2_ESM.pdf]

Dominguez-García & Gómez-Oliva. Supplementary Figure S1

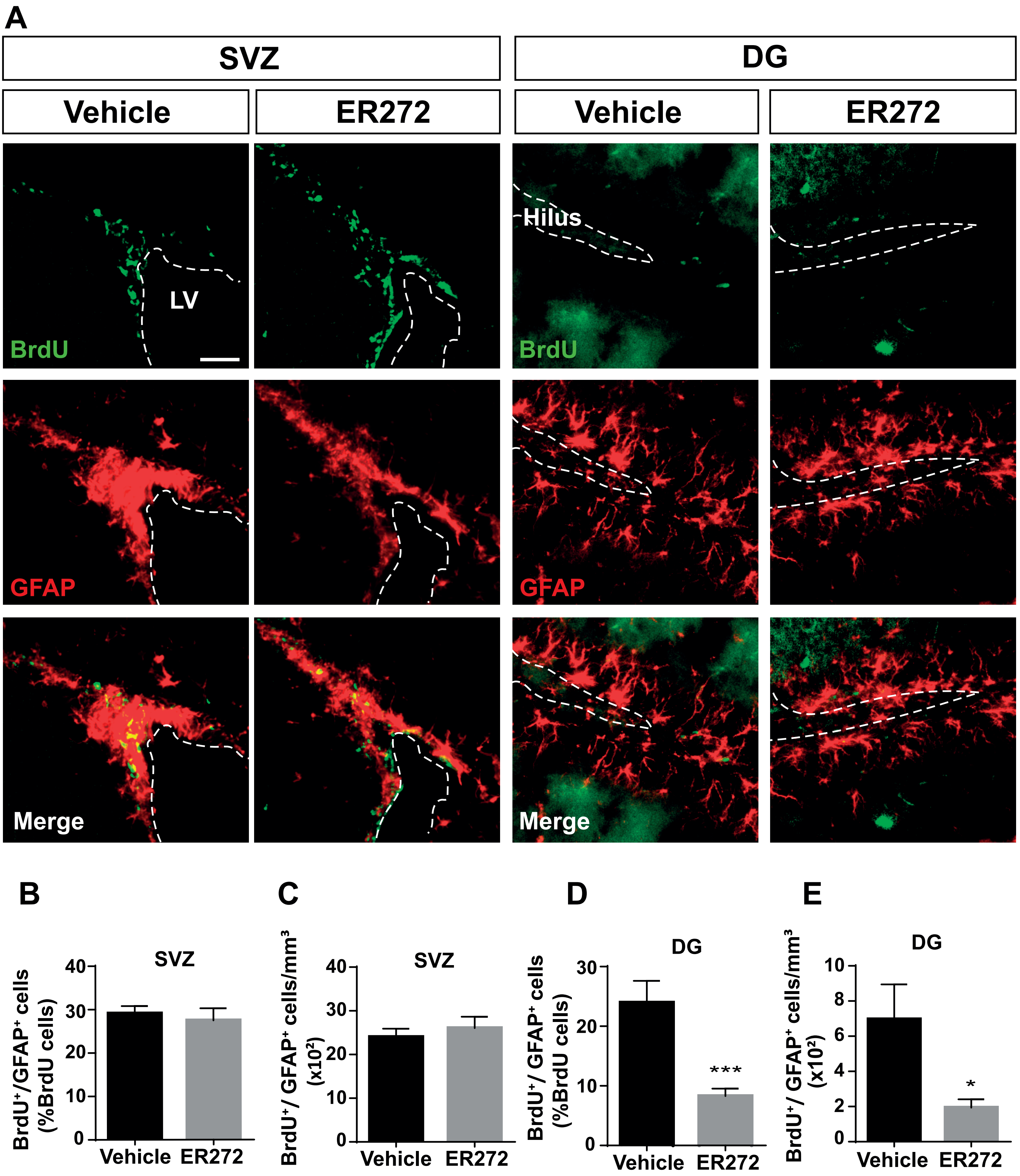

Supplementary Figure S1: Effect of the intranasal administration of ER272 on glial cells within the SVZ and the DG. Intranasal administration of ER272 (1 µM) or only vehicle was performed during 7 days to healthy 2 month-old adult mice. All mice were intraperitoneally-injected with BrdU (100 mg/kg) the last day of treatment as described in methods. **A.** Representative confocal microcopy images of the SVZ and the DG of the hippocampus of adult mice treated with ER272 or only vehicle. Scale bar= 50 µm. The dotted lines indicate lateral ventricle limits (LV) and the DG limits. Slides were processed for the immunohistochemical detection of the proliferation marker BrdU and the glial marker GFAP. **B.** Graph shows the percentage of $BrdU^+$ cells that co-express the glial marker GFAP in the SVZ. Data are the means ± S.E.M of six animals n = 6. No statistically significant differences were found. **C.** Graph shows the number of $BrdU^+$ cells that co-express GFAP per mm^3 in the SVZ. Data are the means ± S.E.M of six animals n = 6. No statistical differences were found. **D.** Graph shows the percentage of $BrdU^+$ cells that co-express the glial marker GFAP in the DG of the hippocampus. Data are the means ± S.E.M of six animals n = 6. Statistical analysis: *** p = 0.0066 in two tailed unpaired Student's t-test comparing ER272 treatment with the control group (treated only with vehicle). **E.** Graph shows the number of $BrdU^+$ cells that co-express GFAP per mm^3 in the DG of the hippocampus. Data are the means ± S.E.M of six animals n = 6. Statistical analysis: * p = 0.0279 in two tailed unpaired Student's t-test comparing ER272 treatment with the control group (treated only with vehicle).

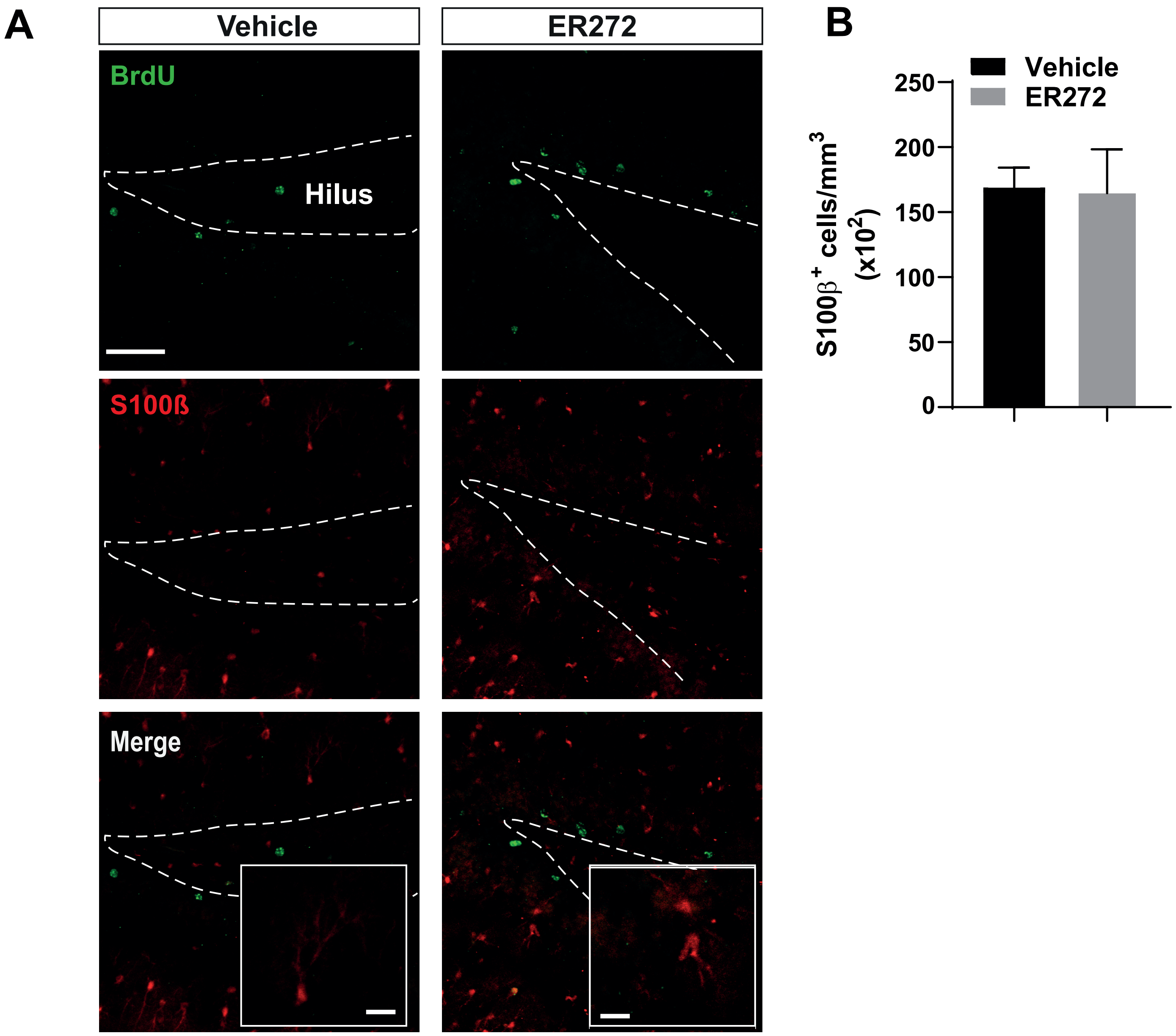

Supplementary Figure S2: Long term intranasal administration of ER272 effect on mature glia cells within the DG of the hippocampus. Intranasal ER272 (1 µM) or only vehicle was administered during 28 days to healthy 2 months-old adult mice. All mice were intraperitoneally-injected with BrdU (100 mg/kg) during the first 14 days every two days as described in methods. **A.** Representative confocal microcopy images of the DG of adult mice hippocampus treated with ER272 or only vehicle. Scale bar= 50 µm. The dotted lines indicate DG limits. Slides were processed for the immunohistochemical detection of the proliferation marker BrdU and the mature glia marker S100β. **B.** Graph shows the number mature glia marked with $S100\beta^{+}$ per mm^3 in the DG of the indicated animal groups. No co-expression of BrdU and S100β wasfound. Data are the means ± S.E.M of 10 animals n = 10. No statistical differences were found.

Dominguez-García & Gómez-Oliva. Supplementary Figure S3

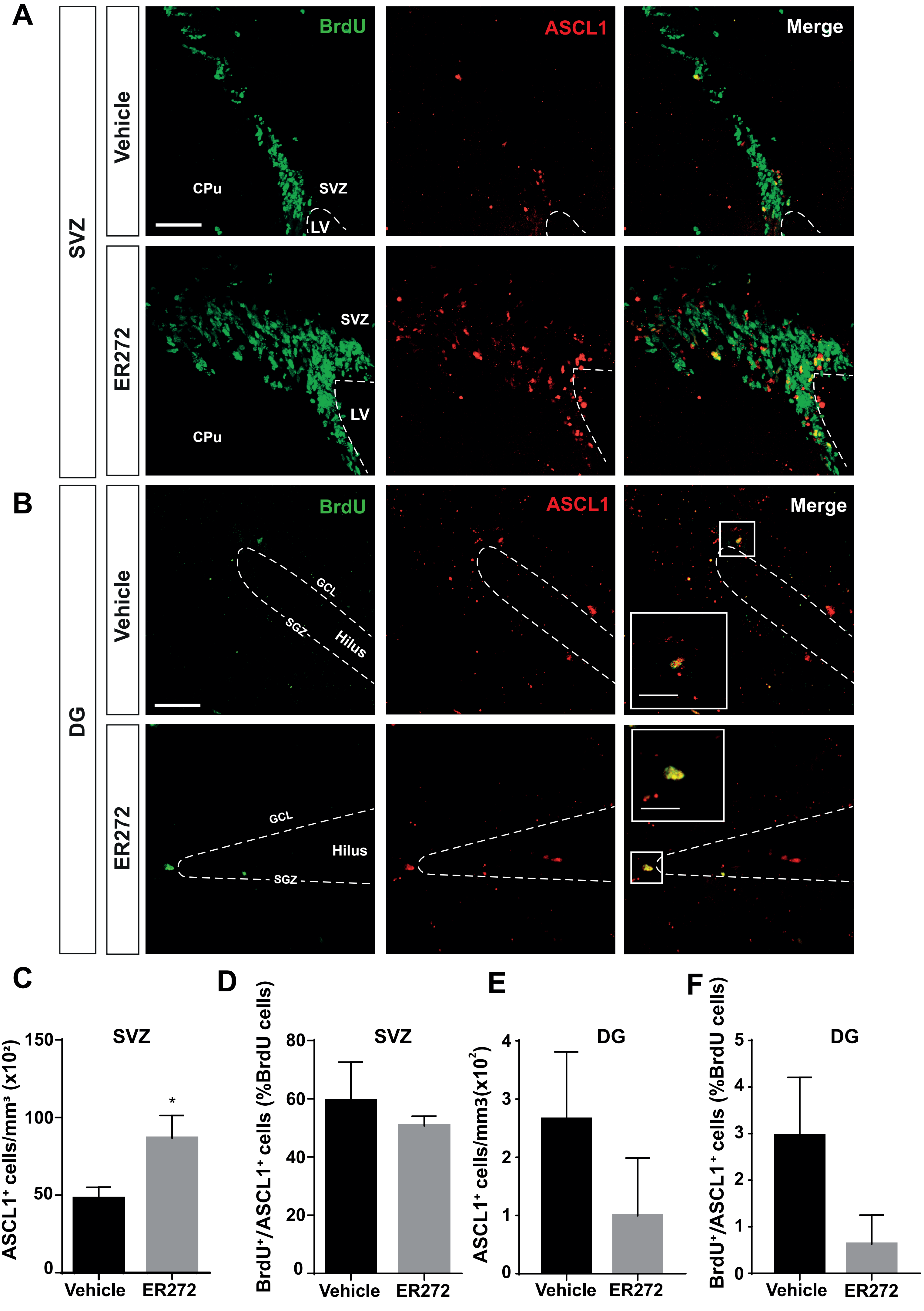

Supplementary Figure S3: Effect of the intranasal administration of ER272 on ASCL$^+$ cells within SVZ and the DG. Intranasal administration of ER272 (1 µM) or only vehicle was performed during 7 days to healthy 2 months-old adult mice. All mice were intraperitoneally injected with BrdU (100 mg/kg) the last day of treatment as described in methods. **A-B** Representative confocal microcopy images of the SVZ and the DG of the hippocampus of adult mice treated with ER272 or only vehicle. Scale bar= 50 µm and 15 µm in high magnification pictures. The dotted lines indicate lateral ventricle limits (LV) and the DG limits. Slides were processed for the immunohistochemical detection of the proliferation marker BrdU and ASCL1. **C.** Graph shows the number of ASCL1$^+$ cells per mm^3 in the SVZ. Data are the means ± S.E.M of six animals n = 6. Statistical analysis: p = 0.040 in two tailed unpaired Student's t-test comparing ER272 treatment with the control group (treated only with vehicle). **D.** Graph shows the percentage of BrdU$^+$ cells that co-express the TAPs marker ASCL1 in the SVZ. Data are the means ± S.E.M of six animals n = 6. No statistical differences were found. **E.** Graph shows the number of ASCL1$^+$ cells in the DG of the hippocampus per mm^3. Data are the means ± S.E.M of six animals n = 6. No statistical differences were found**. F.** Graph shows the percentage of BrdU$^+$ cells that co-express ASCL1 in the DG of the hippocampus. Data are the means ± S.E.M of six animals n = 6. No statistical differences were found.

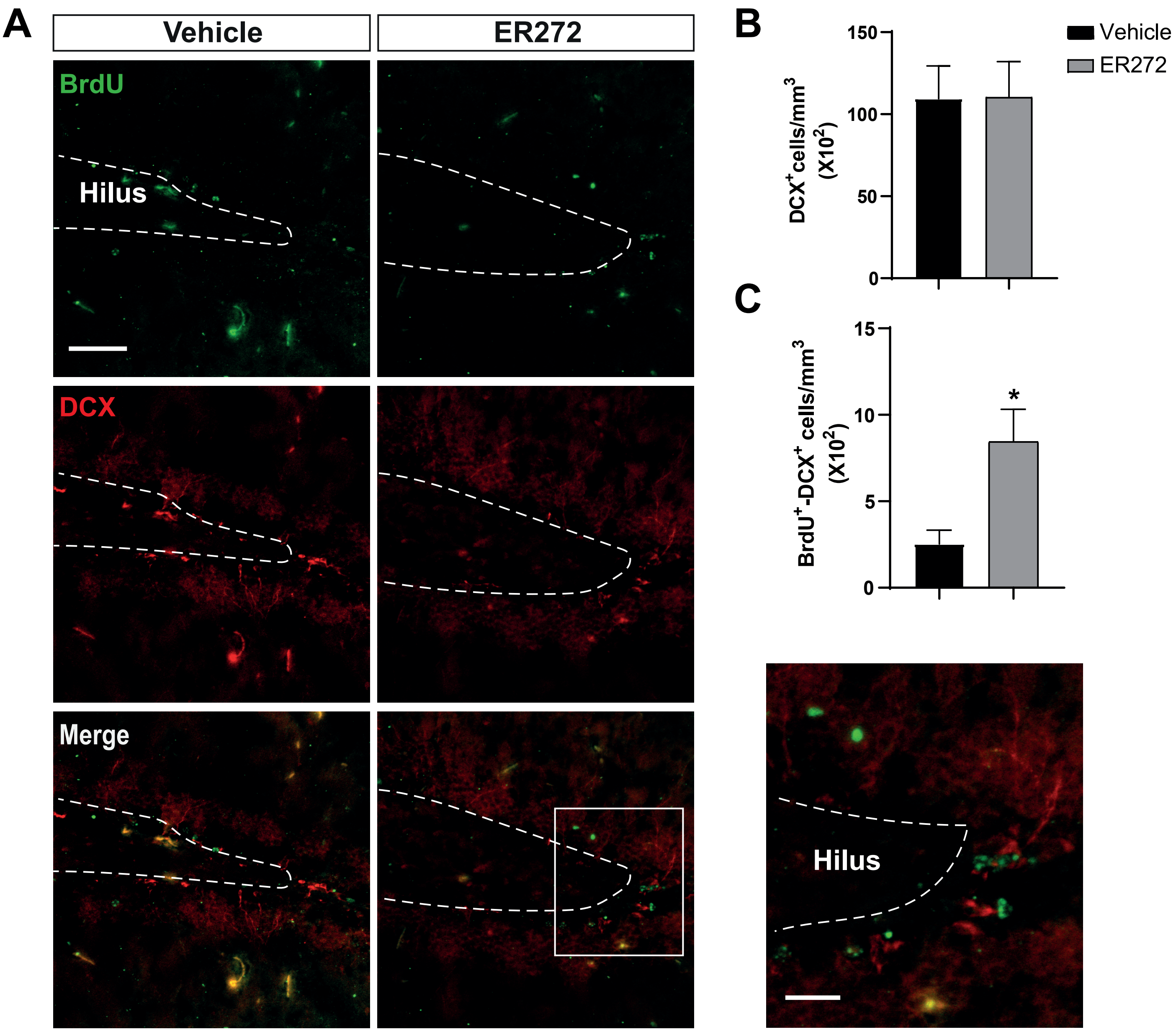

Supplementary Figure S4: Long term intranasal administration of ER272 effect on DCX$^+$ neuroblast cells within the DG of the hippocampus. Intranasal ER272 (1 µM) or only vehicle was administered during 28 days to healthy 2 months-old adult mice. All mice were intraperitoneally-injected with BrdU (100 mg/kg) during the first 14 days every two days as described in methods. **A.** Representative confocal microcopy images of the DG of adult mice hippocampus treated with ER272 or only vehicle. Scale bar = 100 µm and 50 µm in high magnification picture. The dotted lines indicate DG limits. Slides were processed for the immunohistochemical detection of the proliferation marker BrdU and the neuroblast marker DCX. B. Graph shows the number of neuroblast DCX$^+$ per mm^3 in the DG of the indicated animal groups. **C.** Graph shows the number of DCX$^+$ neuroblast that co-express the proliferation marker BrdU per mm^3. * $p = 0.0198$ in two tailed unpaired Student's t-test comparing ER272 treatment vs. only vehicle infused mice. Data are the means ± S.E.M of 10 animals n = 10. No statistical differences were found.

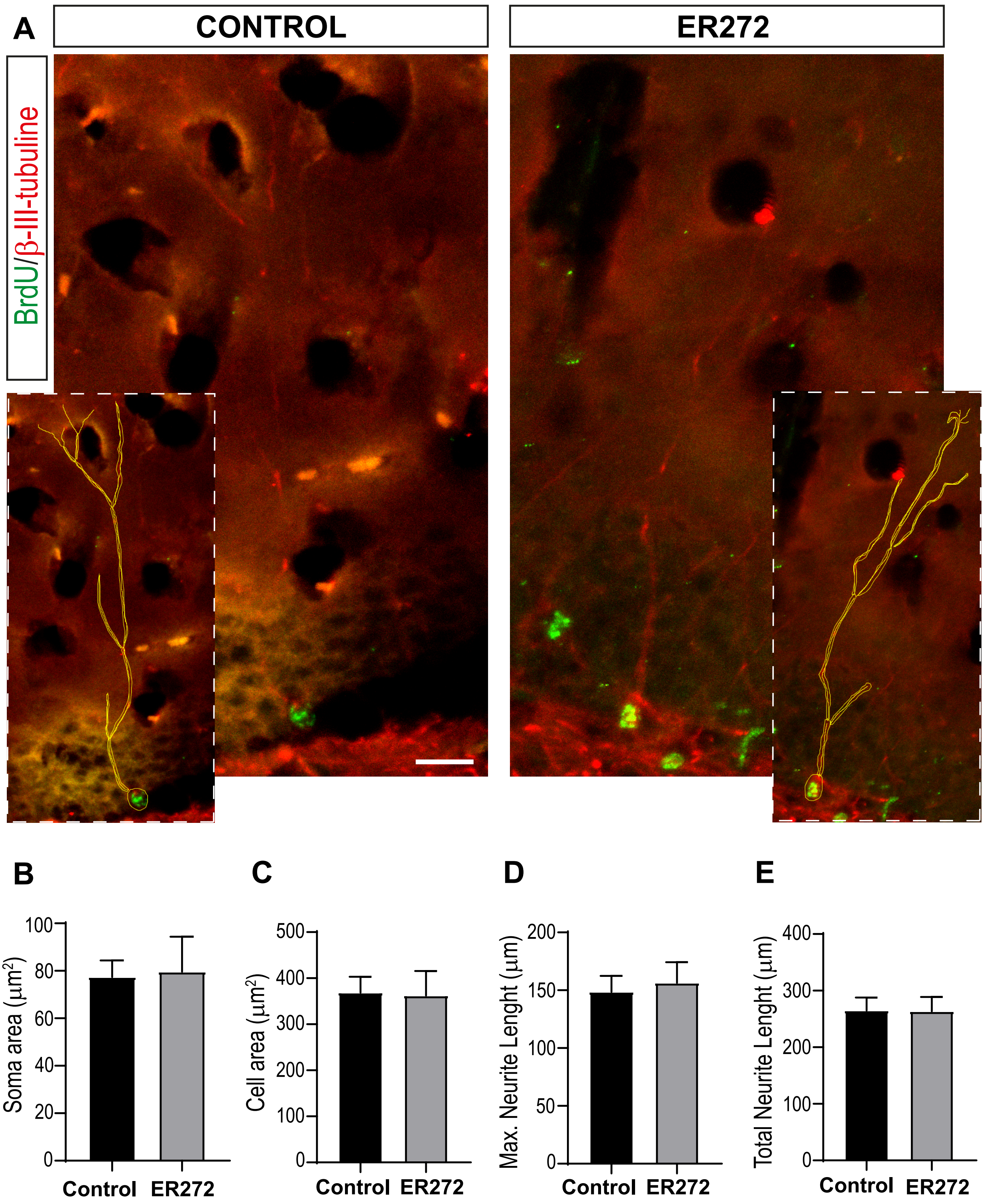
A
CONTROL
ER272
BrdU/β-III-tubuline
B
Soma area (μm2)
100
80
60
40
20
0
Control
ER272
C
Cell area (μm2)
500
400
300
200
100
0
Control
ER272
D
Max. Neurite Lenght (μm)
200
150
100
50
0
Control
ER272
E
Total Neurite Lenght (μm)
400
300
200
100
0
Control
ER272

Supplementary Figure S5: Effect of long term intranasal administration of ER272 on mature neuron (BrdU$^+$/β–III-tubuline$^+$) morphology (pilot study). Intranasal ER272 (1 μM) or only vehicle was administered during 28 days to healthy 2 month-old adult mice. All mice were intraperitoneally-injected with BrdU (100 mg/kg) during the first 14 days every two days as described in methods. **A.** Visual comparison of mature BrdU$^+$/β–III-tubuline$^+$ neurons of the DG of adult mice treated with ER272 or only vehicle. Slides were processed for the immunohistochemical detection of the proliferation marker BrdU and the neuronal marker β–III-tubuline. BrdU$^+$/β–III–tubuline$^+$ cells were manually restored with ImageJ software. Scale bar = 15 μm. **B.** Soma area (μm^2) of BrdU$^+$/β-III-tubuline$^+$ cells in the DG of the indicated animal groups; no differences were detected ($p = 0.911$) using Student's t-test. **C.** Cell area (μm^2) of BrdU$^+$/β–III-tubuline$^+$ cells in the DG of the indicated animal groups; no differences were detected ($p = 0.931$) using Student's t-test. **D.** Maximum neurite length (μm) of BrdU$^+$/β–III–tubuline$^+$ cells in the DG of the indicated animal groups; no differences were detected ($p = 0.758$) using Student's t-test. **E.** Total neurite length (μm) of BrdU$^+$/β–III-tubuline$^+$ cells in the DG of the indicated animal groups; no differences were detected ($p = 0.972$) using Student's t-test. At least 10 neurons/condition were assessed. Data are the means ± S.E.M of 6 animals, $n = 6$.

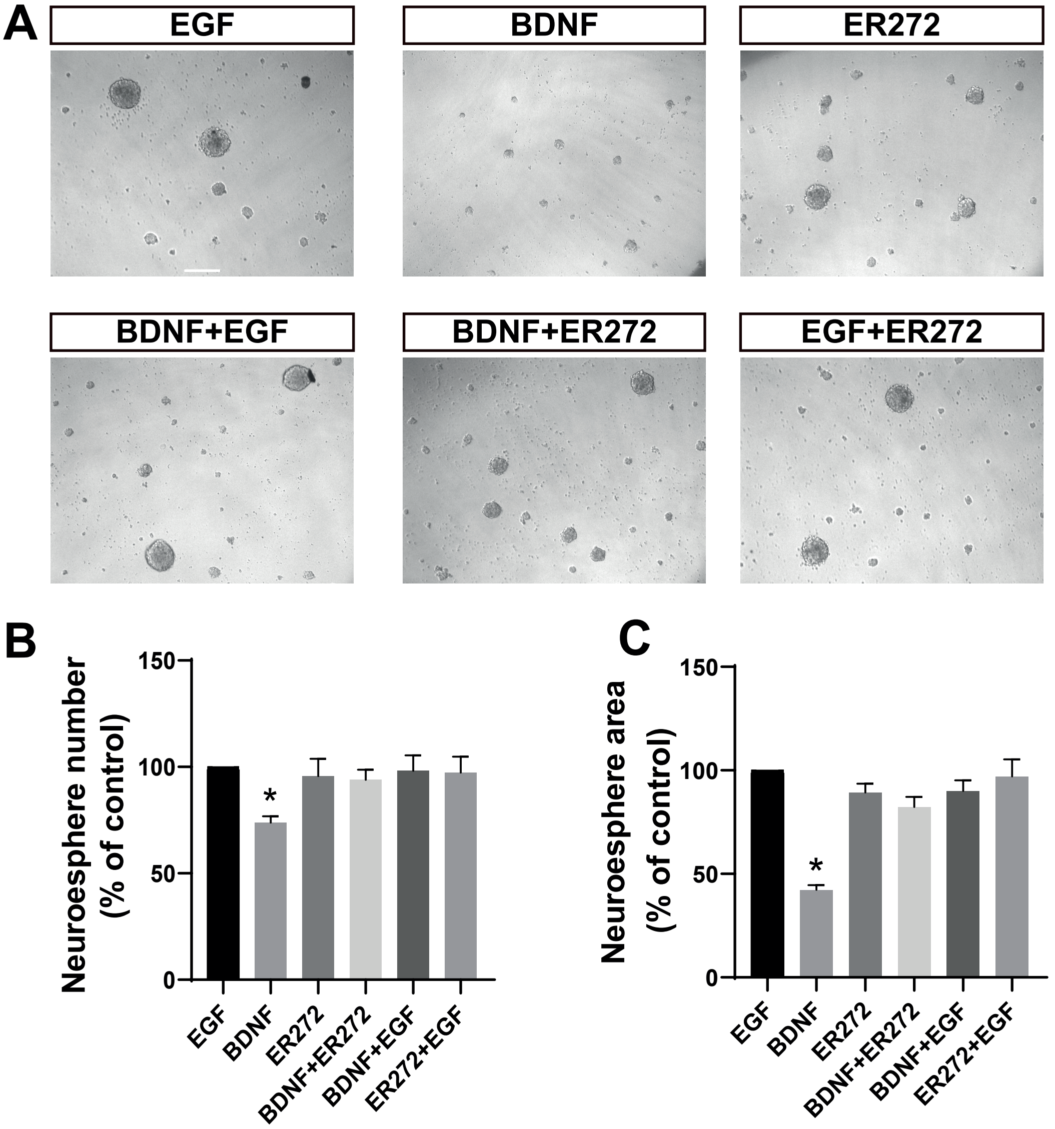

Supplementary Figure S6: The BDNF growth factor treatment has not an additive effect with EGF or ER272 in vitro. A. Representative phase-contrast microscopy images of neurospheres cultured for 72 h in presence or absence of the indicated growth factors and ER272. Scale bar indicates 100 µm. **B.** Graph shows the effect of the different treatments on neurospheres number expressed as the percentage of control (only EGF treatment). Data shown are the mean ± S.E.M. of 9 independent measurements. Statistical analysis: *$p = 0.0035$ in two tailed unpaired Student's t-test comparing EGF treatment with BDNF treatment. *$p = 0.0131$ in two tailed unpaired Student's t-test comparing ER272 treatment with BDNF treatment. *$p = 0.0211$ in two tailed unpaired Student's t-test comparing ER272+BDNF treatment with BDNF treatment. *$p = 0.0062$ in two tailed unpaired Student's t-test comparing EGF+BDNF treatment with BDNF treatment. *$p = 0.0079$ in two tailed unpaired Student's t-test comparing EGF+ER272 treatment with BDNF treatment. **C.** Graph shows the effect of the different treatments on neurospheres area expressed as the percentage of control (only EGF treatment). Data shown are the mean ± S.E.M. of 9 independent measurements. Statistical analysis: *$p < 0.0001$ in two tailed unpaired Student's t-test comparing EGF treatment with BDNF treatment. *$p < 0.0001$ in two tailed unpaired Student's t-test comparing ER272 treatment with BDNF treatment. *$p < 0.0001$ in two tailed unpaired Student's t-test comparing ER272+BDNF treatment with BDNF treatment. *$p < 0.0001$ in two tailed unpaired Student's t-test comparing EGF+BDNF treatment with BDNF treatment. *$p < 0.0001$ in two tailed unpaired Student's t-test comparing EGF+ER272 treatment with BDNF treatment.

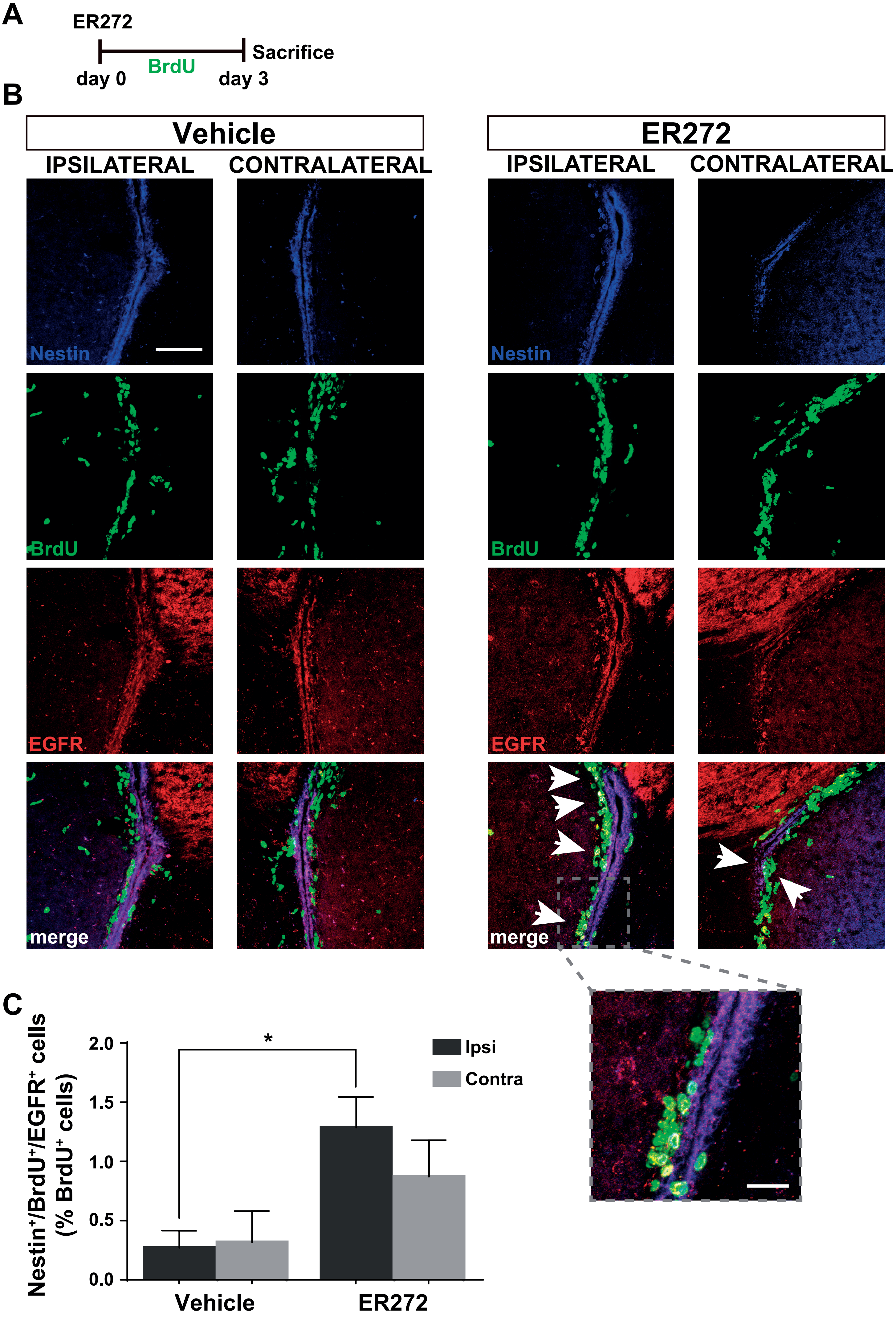
A
ER272
Sacrifice
BrdU
day 0
day 3
B
Vehicle
ER272
IPSILATERAL
CONTRALATERAL
IPSILATERAL
CONTRALATERAL
Nestin
BrdU
EGFR
merge
Nestin
BrdU
EGFR
merge
C
Nestin⁺/BrdU⁺/EGFR⁺ cells (% BrdU⁺ cells)
2.0
1.5
1.0
0.5
0.0
*
Ipsi
Contra
Vehicle
ER272

Supplementary Figure S7: Effect of the intracerebroventricular administration of ER272 on the number of proliferative $EGFR^+$ cells within the SVZ. A. Intracerebroventricular (ICV) administration of ER272 (1 µM) or only vehicle and BrdU injections was performed in healthy mice as described in methods. **B.** Confocal representative images show adult mice coronal sections including the SVZ ipsilateral and contralateral processed for immunohistochemical detection of the proliferation marker BrdU, the undifferentiated neural progenitor marker nestin and the epidermal growth factor receptor EGFR. Scale bar represents 25 µm and 10 µm in the high magnification pictures. **C.** Graph shows the percentage of $BrdU^+$ cells that co-express nestin and EGFR in the ipsilateral and the contralateral SVZ in treated and vehicle administrated mice. Data are the means ± S.E.M of six animals n = 6. Statistical analysis: *p = 0.0262 in two tailed unpaired Student's t-test comparing ER272 treatment with the control group (treated only with vehicle) in the ipsilateral site.

Dominguez-García & Gómez-Oliva. Supplentary Figure S8

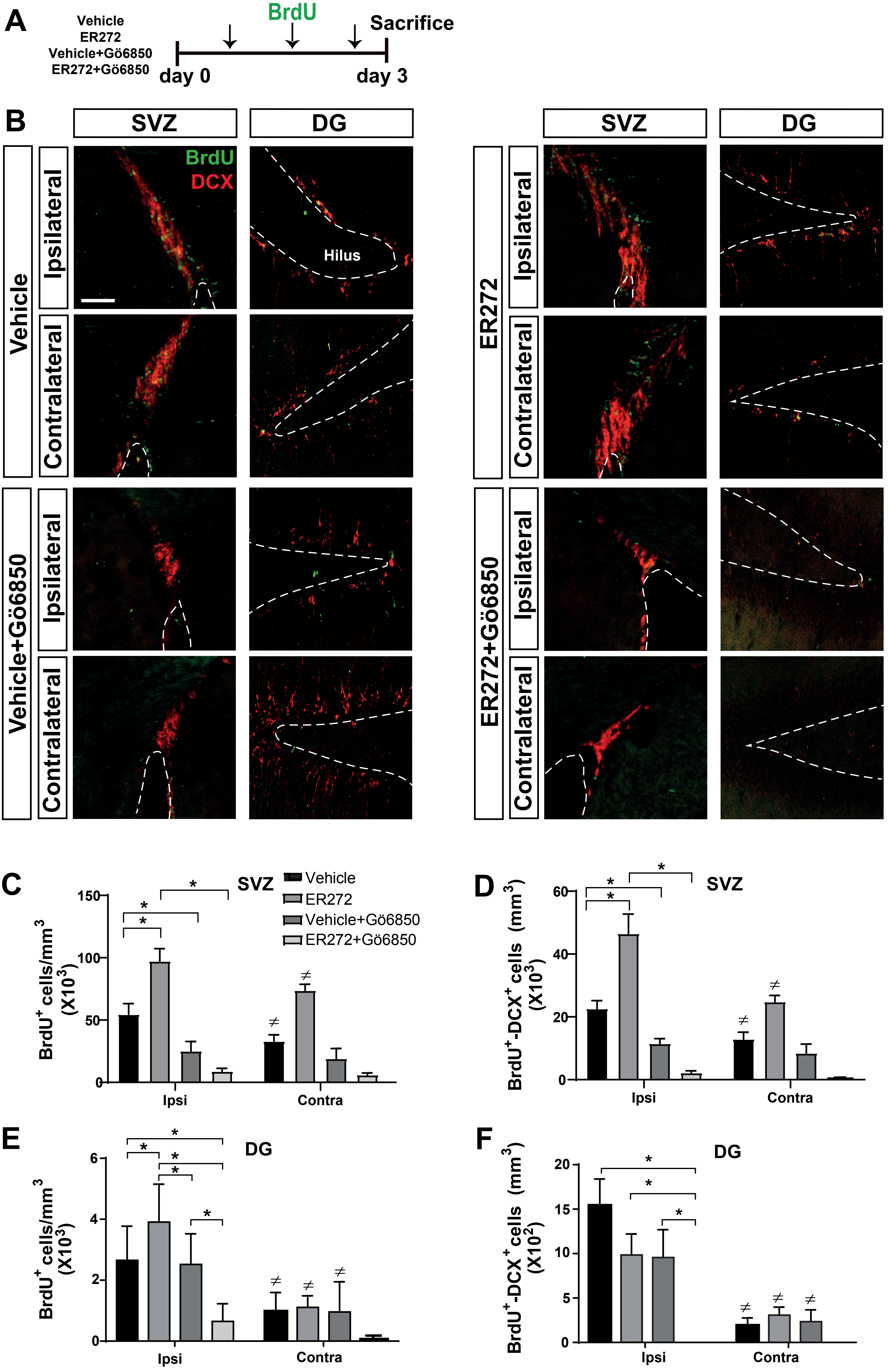

Supplementary Figure S8: The general PKC inhibior Gö6850 reverts ER272 effects in vivo: Intracerebroventricular (ICV) administration of vehicle, ER272 (1µM), the general PKC inhibitor Gö6850 (1 µM) or a combination of ER272 and Gö6850 was performed in a single injection to healthy 2 month-old adult mice. All mice were intraperitoneally-injected with BrdU (100 mg/kg) during 3 days as described in methods. **A.** Scheme of BrdU administration and treatments. **B.** Representative confocal microcopy images of the SVZ and the DG of the hippocampus of adult mice treated with vehicle, ER272 (1 µM), the general PKC inhibitor Gö6850 (1 µM) or a combination of ER272 and Gö6850. Scale bar = 100 µm. The dotted lines indicate lateral ventricle limits (LV) and the DG limits. Slides were processed for the immunohistochemical detection the proliferation marker BrdU and the neuroblast marker DCX.. **C.** Graph shows the number of BrdU$^+$ cells per mm^3 in the SVZ comparing ipsilateral and contralateral site. Data are the means ± S.E.M of six animals n = 6. Statistical analysis: # p = 0.003 and # p = 0.015; comparing ipsilateral and contralateral site in vehicle injected mice and ipsilateral and contralateral site in ER272 injected mice respectively. * p = 0.0128 comparing ipsilateral vehicle injected mice with ipsilateral ER272 injected mice. * p = 0.046 comparing ipsilateral vehicle injected mice with vehicle+Gö6850 injected mice. * p<0.0001 comparing ipsilateral ER272 injected mice with ER272+Gö6850 injected mice. **D.** Graph shows the number of BrdU$^+$ cells that co-express the neuroblast marker DCX per mm^3 in the SVZ comparing ipsilateral and contralateral site. Data are the means ± S.E.M of six animals n = 6. Statistical analysis: # p = 0.001 and # p = 0.0083 comparing ipsilateral and contralateral site in vehicle injected mice, and ipsilateral and contralateral site in ER272 injected mice respectively. * p = 0.0064 comparing ipsilateral vehicle injected mice with ipsilateral ER272 injected mice. * p = 0.0227 comparing ipsilateral vehicle injected mice with vehicle+Gö6850 injected mice. * p = 0.0004 comparing ipsilateral ER272 injected mice with ER272+Gö6850 injected mice. **E.** Graph shows the number of BrdU$^+$ cells per mm^3 in the DG comparing ipsilateral and contralateral site. Data are the means ± S.E.M of six animals n = 6. Statistical analysis: # p = 0.001, # < 0.001 and # p = 0.002 comparing ipsilateral and contralateral site in vehicle injected mice, ipsilateral and contralateral site in ER272 injected mice, and ipsilateral and contralateral site in ER272+Gö6850 respectively. * p = 0.0228 comparing ipsilateral vehicle injected mice with ipsilateral ER272 injected mice. * p = 0.0001 comparing ipsilateral vehicle injected mice with ER272+Gö6850 injected mice. * p = 0.0122 comparing ipsilateral ER272 injected mice with vehicle+Gö6850 injected mice. * p < 0.0001 comparing ipsilateral ER272 injected mice with ER272+Gö6850 injected mice. * p = 0.0003 comparing ipsilateral vehicle+Gö6850 injected mice with ER272+Gö6850 injected mice. **F.** Graph shows the number of BrdU$^+$ cells that co-express the neuroblast marker DCX per mm^3 in the DG comparing ipsilateral and contralateral site. Data are the means ± S.E.M of six animals n = 6. Statistical analysis: # p < 0.0001, # p = 0.003 and # p = 0.0052, comparing ipsilateral and contralateral site in vehicle injected mice, ipsilateral and contralateral site in ER272 injected mice, and ipsilateral and contralateral site vehicle+Gö6850 injected mice respectively. Statistical analysis: * p < 0.0001, * < 0.0001 and * p = 0.0004 comparing ipsilateral ER272+Gö6850 with ipsilateral vehicle, ER272 and vehicle+Gö6850 in vehicle injected mice respectively. All statistical analyses were performed using a two tailed unpaired Student's t-test.
